# Cross-Species Array Comparative Genomic Hybridization Identifies Novel Oncogenic Events in Zebrafish and Human Embryonal Rhabdomyosarcoma

**DOI:** 10.1371/journal.pgen.1003727

**Published:** 2013-08-29

**Authors:** Eleanor Y. Chen, Kimberly P. Dobrinski, Kim H. Brown, Ryan Clagg, Elena Edelman, Myron S. Ignatius, Jin Yun Helen Chen, Jillian Brockmann, G. Petur Nielsen, Sridhar Ramaswamy, Charles Keller, Charles Lee, David M. Langenau

**Affiliations:** 1 Division of Molecular Pathology, Massachusetts General Hospital, Charlestown, Massachusetts, United States of America; 2 Harvard Stem Cell Institute, Boston, Massachusetts, United States of America; 3 Cancer Center, Department of Medicine, Massachusetts General Hospital, Boston, Massachusetts, United States of America; 4 Harvard Medical School, Boston, Massachusetts, United States of America; 5 Department of Pathology, Brigham and Women's Hospital, Boston, Massachusetts, United States of America; 6 Department of Pathology and Cell Biology, College of Medicine, University of Southern Florida, Tampa, Florida, United States of America; 7 Department of Biology, Portland State University, Portland, Oregon, United States of America; 8 Department of Pathology, Massachusetts General Hospital, Boston, Massachusetts, United States of America; 9 Pediatric Cancer Biology Program, Department of Pediatrics, Oregon Health and Science University, Portland, Oregon, United States of America; Stanford University School of Medicine, United States of America

## Abstract

Human cancer genomes are highly complex, making it challenging to identify specific drivers of cancer growth, progression, and tumor maintenance. To bypass this obstacle, we have applied array comparative genomic hybridization (array CGH) to zebrafish embryonal rhabdomyosaroma (ERMS) and utilized cross-species comparison to rapidly identify genomic copy number aberrations and novel candidate oncogenes in human disease. Zebrafish ERMS contain small, focal regions of low-copy amplification. These same regions were commonly amplified in human disease. For example, 16 of 19 chromosomal gains identified in zebrafish ERMS also exhibited focal, low-copy gains in human disease. Genes found in amplified genomic regions were assessed for functional roles in promoting continued tumor growth in human and zebrafish ERMS – identifying critical genes associated with tumor maintenance. Knockdown studies identified important roles for Cyclin D2 (CCND2), Homeobox Protein C6 (HOXC6) and PlexinA1 (PLXNA1) in human ERMS cell proliferation. PLXNA1 knockdown also enhanced differentiation, reduced migration, and altered anchorage-independent growth. By contrast, chemical inhibition of vascular endothelial growth factor (VEGF) signaling reduced angiogenesis and tumor size in ERMS-bearing zebrafish. Importantly, *VEGFA* expression correlated with poor clinical outcome in patients with ERMS, implicating inhibitors of the VEGF pathway as a promising therapy for improving patient survival. Our results demonstrate the utility of array CGH and cross-species comparisons to identify candidate oncogenes essential for the pathogenesis of human cancer.

## Author Summary

Cancer is a complex genetic disease that is often associated with regional gains and losses of genomic DNA segments. These changes result in aberrant gene expression and drive continued tumor growth. Because amplified and deleted DNA segments tend to span large regions of chromosomes, it has been challenging to identify the genes that are required for continued tumor growth and progression. Array comparative genomic hybridization (array CGH) is an effective technology in identifying abnormal copy number variations in cancer genomes. In this study, array CGH was used in a zebrafish model of embryonal rhabdomyosarcoma - a pediatric muscle tumor. Our work shows that the zebrafish cancer genome contains a small number of recurrent DNA copy number changes, which are also commonly amplified in the human disease. Moreover, these chromosomal regions are small, facilitating rapid identification of candidate oncogenes. A subset of genes identified in zebrafish array CGH was prioritized for functional characterization in human ERMS, identifying evolutionarily conserved pathways that regulate proliferation, migration, differentiation, and neovascularization. Our results demonstrate the broad utility of cross-species array CGH comparisons of human and zebrafish cancer and provide a much needed discovery platform for identifying critical cancer-causing genes in a wide range of malignancies.

## Introduction

Rhabdomyosaroma (RMS) is the most common soft tissue sarcoma of childhood [1] and falls into two major histopathologic subtypes in children - embryonal and alveolar. Embryonal rhabdomyosaroma (ERMS) accounts for approximately 60% of childhood cases and is frequently associated with RAS pathway activation [2]–[5]. Treatment for either RMS subtype requires surgical resection, chemotherapy, and radiation with overall poor prognosis for patients with high-risk features, metastasis, or relapse disease. Thus, there is great interest in elucidating key molecular pathways and genetic factors that are involved in continued RMS growth and tumor maintenance. Cytogenetic studies, including array Comparative Genomic Hybridiation (array CGH), identify frequent but inconsistent gains and losses of whole or partial chromosome arms and rare focal high-level amplifications in both human ERMS and ARMS [5]–[9], largely precluding the identification of specific drivers of cancer in this disease. Moreover, array CGH and cross-species comparisons between mouse and human RMS have largely failed to identify functionally important genes contained within common copy number alterations (CNAs). In one report, RMS that arose in *Ptch1* ***^+^*** ^*/****−***^ *Blm^tm3Brd/tm3Brd^* (a hypomorphic *Blm* allele) mice exhibited a gain of chromosome 10 in 80% of cases [10], but the oncogenes associated with this chromosomal gain remain undefined due to the large number of candidate genes found within this region. Moreover, extension of these findings to human RMS has not been reported. Rubin et al. recently showed that greater than 30% of ERMS arising in mice that harbor *p53* homozygous deletion and/or *Ptch1* heterozygous deletion lack a defined molecular signature or genetic lesion, suggesting undiscovered pathways likely contribute to ERMS transformation, growth, and tumor maintenance [11]. To date, there remains a need for novel gene discovery methods to identify genes and pathways essential for tumor growth, progression, and maintenance in human cancer – including ERMS.

Zebrafish cancer shares molecular and pathological similarities to human disease [4], [12]–[16]. For example, Lam et al. (2006) was the first to use comparative analysis of microarray data from zebrafish and human liver tumors to demonstrate a conserved molecular profile during tumor progression [13]. Building on this work, microarray gene expression studies of zebrafish ERMS and cross-species comparison to human disease identified RAS pathway activation as a common initiating event in zebrafish and human ERMS. Activating RAS mutations have also been identified in numerous studies of human ERMS [2]–[5], [17]. Most recently, Paulson et al reported that 11 of 26 (42%) human ERMS samples harbored activating RAS mutations along with acquisition of additional CNAs as detected array CGH [5], suggesting that additional genetic lesions are likely required to drive oncogenic transformation to ERMS. Not surprisingly, zebrafish cancers also exhibit recurrent chromosomal gains and losses similar to those found in human cancer. For example, transgenic models of zebrafish melanoma, T-cell acute lymphoblastic leukemia (T-ALL), and ERMS contain genomic imbalances including high-level gains and losses [18]. However, specific driver events could not be identified in these studies due to the low resolution of this platform. Using high-resolution array CGH, Zhang et al (2010) demonstrated the aneuploid nature of zebrafish malignant nerve sheath tumors (MPNST), a feature that also characterizes the human disease, and identified a subset of genes that are co-amplified as high-copy gains in human MPNST [19]. High-resolution array CGH has also been applied to zebrafish T-ALL and identified a subset of genes contained within CNAs that were also amplified or deleted in human disease [20]. These latter two studies have demonstrated the utility of array CGH technology in detecting copy number aberrations and candidate driver genes in zebrafish tumor models, yet functional relevance of identified genes in human disease has not been reported nor have these genes been assessed for roles in regulating tumor maintenance – providing novel targets for therapy in established tumors.

Capitalizing on a zebrafish model of *kRASG12D*-induced ERMS that shares common histopathological, genetic, and molecular characteristics of human ERMS [4], [21], [22], we have utilized high-resolution array CGH to identify novel CNAs in ERMS. Remarkably, our array CGH analysis revealed focal CNAs that span short genomic regions and contain only 1–3 genes. To validate the functional significance of amplified genes in human ERMS, we prioritized six genes for initial characterization in human ERMS cell lines. Of these six genes, gene knockdown of *Cyclin D2 (CCND2), Homeobox C6 (HOXC6), PlexinA1* (PLXNA1) inhibited proliferation of human ERMS. PLXNA1 also exhibited important roles in blocking ERMS cells in early stages of muscle differentiation, enhancing migration, and altering anchorage-independent growth. CCND2, HOXC6, PLXNA1, and Vascular Endothelial Growth Factor A (VEGFA) were also highly expressed in a large fraction of human primary RMS, supporting prominent roles for these genes in rhabdomyosarcoma. Chemical inhibition of VEGF signaling reduced tumor growth *in vivo* with an associated decrease in angiogenesis, implicating VEGF inhibitors as promising therapeutic agents for ERMS. Taking advantage of tractable features of zebrafish cancer genomes such as smaller CNA intervals and regions of conserved homology with human disease, our study demonstrates the effective use of array CGH to identify oncogenes required for continued tumor growth in human rhabdomyosaroma, providing novel therapeutic targets for the treatment of ERMS.

## Results

### Zebrafish array CGH identifies novel and conserved CNAs in human ERMS

**Figure 1:**
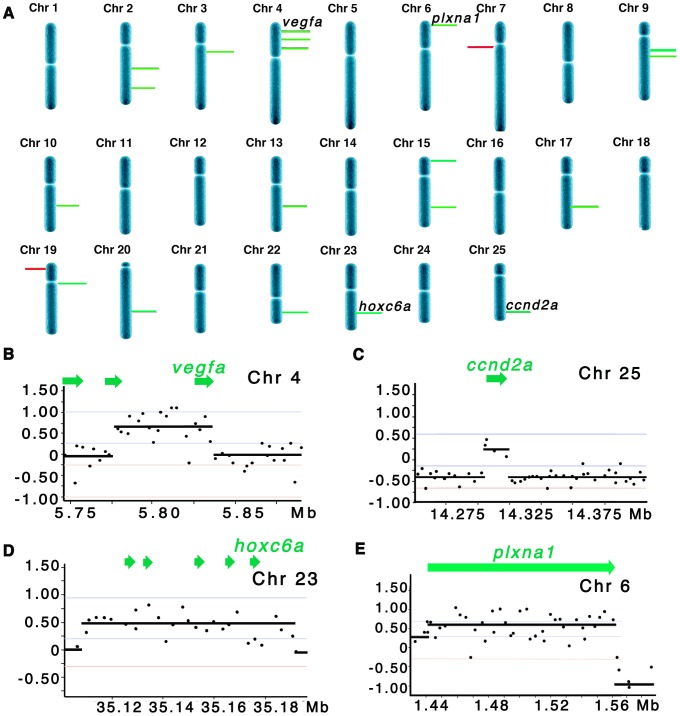
Array CGH reveals cancer-specific chromosomal abnormalities in zebrafish ERMS. (A) Summary of common gene-containing CNA gains (green) and losses (red) in 20 animals examined. Only recurrent CNAs found in ≥3 samples are shown. The height of each bar correlates with the frequency of each aberration. Detailed view of regional gains for *vegfa* on chromosome 4 (B), *ccnd2a* on chromosome 25 (C), *hoxc6a* on chromosome 23 (D), and *plxna1* on chromosome 6 (E). Y-axis denotes log2 ratio of the probes and X-axis denotes genomic coordinates.

Array CGH was performed on genomic DNA isolated from twenty *kRASG12D*-induced zebrafish ERMS and compared to adjacent normal tissue. Array CGH revealed a complex CNA pattern with relative gains being observed more frequently than losses. For example, we identified 190 regions of amplification and 35 deletions recurrent in ≥3 zebrafish tumors analyzed (Table S1). Remarkably, only 2 of 20 zebrafish samples exhibit evidence of aneuploidy, contrasting starkly with human ERMS where nearly all human RMS harbored regions of extensive aneuploidy [5]. While 10 zebrafish ERMS showed evidence for CNAs in coding regions of the genome, only 3 exhibited a high frequency of multiple gains (Table 1 and S1). In total, we identified 19 gains and 2 losses in gene-containing amplicons that were recurrent in at least three zebrafish ERMS samples. Candidate genes in these regions were predominantly amplified as low-level gains, which averaged 1–3 genes and spanned only 48+/−27 kb (+/− SD, Table 1; Fig. 1A). Copy number alterations were validated by qPCR of genomic DNA (Fig. S1).

**Table 1:** Comparison of array CGH analyses in zebrafish and human ERMS.

Zebrafish Chr	Start	End	Size (bp)	Samples with CNA	Genes in Interval	Human Gene Homologues	Chr	Start	End	Size (bp)	with Amplified CNA	Genes in interval
2	3.2E+07	3.2E+07	31975	4 of 20	patched 2 (ptch2)	PTCH2	1	45022354	45089190	66836	1 of 26	9
2	4.6E+07	4.6E+07	21677	4 of 20	mbnl1	MBNL1	3	153448149	153676279	228130	2 of 26	2
3	2.1E+07	2.1E+07	72013	3 of 20	hoxb3a, hoxb4a, hoxb5a,	HOXB3, HOXB4, HOXB5	17	43902466	44075242	172776	5 of 26	7
					hoxb6a, hoxb7a, hoxb8a,	HOXB6, HOXB7, HOXB8						
					hoxb9a, mir10c	HOXB9						
4	5776500	5833215	56715	3 of 20	rsph9, mrps18a, **vegfa**	VEGFA	6	43803475	43880447	76972	6 of 26	2
4	1.2E+07	1.2E+07	85059	3 of 20	**braf**, cry1a, tnnt1	BRAF	7	139802093	140482012	679919	5 of 26	7
						CRY1A	12	105791516	106162074	370558	9 of 26	4
						TNNT1	19	60246821	60592535	345714	8 of 26	25
4	1.8E+07	1.8E+07	41361	3 of 20	apaf1	APAF1	12	96951092	97889931	938839	10 of 26	10
6	1563960	1584371	20411	4 of 20	**plxna1**	PLXNA1	3	128138430	128299614	161184	4 of 26	3
9	2.4E+07	2.4E+07	24615	6 of 20	stk17b	STK17B	2	196716864	196782315	65451	10 of 26	2
9	2.4E+07	2.4E+07	32894	6 of 20	tmeff2a	TMEFF2	2	(192522992)	(192767889)	244897	11 of 26	Aneuploid
10	1.8E+07	1.9E+07	70481	3 of 20	pcdh1g11, pcdh1gb9,	PCDHGC5	5	140848162	140948899	100737	5 of 26	7
					pcdh1g9, pcdh1gb2,							
					pcdh1g3, pcdh1g18							
13	3.4E+07	3.4E+07	16915	6 of 20	smap1	SMAP1	6	71434200	71628437	194237	0 of 26	Not Amplified
15	2.1E+07	2.1E+07	50974	5 of 20	aldh3a2	ALDH3A	17	19492656	19521500	28844	0 of 26	Not Amplified
15	186438	238755	52317	6 of 20	**limk1**	LIMK1	17	72888258	73599600	711342	7 of 26	9
17	2.1E+07	2.1E+07	82203	3 of 20	ppp1r3ca	PPP1R3C	10	92581714	93961386	1E+06	1 of 26	17
19	1.4E+07	1.4E+07	29638	4 of 20	hoxa5a, hoxa4a,	HOXA1, HOXA2, HOXA3	7	27095146	27150002	54856	7 of 26	5
					hoxa3a, hoxa1a	HOXA4, HOXA5						
20	4.6E+07	4.6E+07	69342	3 of 20	tram2	TRAM2	6	52500302	52578833	78531	0 of 26	3
22	3E+07	3E+07	87467	3 of 20	acbd4, hexim1	ACBD4, HEXIM1	17	40559338	40681751	122413	2 of 26	3
23	3.5E+07	3.5E+07	94371	3 of 20	hoxc3a, hoxc4a, mir10b-2,	HOXC13, HOXC12, HOTAIR,	12	52461084	52737632	276548	12 of 26	10
					hoxc5a, **hoxc6a**, hoxc8a,	HOXC11, HOXC10, HOXC9,						
					hoxc9a, mir196a-1, hoxc10a,	HOXC8, HOXC6, HOXC5						
					hoxc11a, hoxc12a, hoxc13a	HOXC4						
25	1.4E+07	1.4E+07	20581	5 of 20	**ccnd2a**, tigara	CCND2	12	3866904	4377212	510308	8 of 26	3
									Average:	328,193+/−278,351		7.3
**ERMS Deleted**												
19	3289509	3305879	16370	3 of 20	illr3	No homologue in Human						
7	1.7E+07	1.7E+07	42336	3 of 20	nitr1h, nitr1j	No homologues in Human						

Zebrafish gene-containing, CNAs were compared to those identified in 26 primary human ERMS samples by Paulson et al. (2011). Genes selected for characterization in this study are in bold.

To assess whether CNAs identified in zebrafish ERMS were conserved in human disease, zebrafish array CGH data was compared to the high-resolution array CGH data from 26 human ERMS samples [5]. Of the 26 samples, 11 carried activating RAS mutations as assessed by Sanger sequencing analysis [5]. The two regions of chromosomal loss in zebrafish ERMS contained zebrafish-specific genes that failed to have human homologues (Table 1). By contrast, genes contained within the 19 CNA gains mapped to 21 distinct homologous genomic regions in human. Utilizing the same statistical algorithms and threshold settings as outlined by Paulson et al [5], we discovered that 18 of 21 homologous regions were also amplified as low-copy gains in human ERMS samples (Table 1). Like zebrafish ERMS, these CNAs were focal, low-copy gains spanning 328 kB+/−278 kB and contained 7.3 genes on average (Table 1 and S1).

To demonstrate the efficacy of our array CGH approach to identify evolutionarily conserved oncogenes essential for driving tumor progression and maintenance, we prioritized CNAs that contained genes and were amplified in both zebrafish and human ERMS. In total, six candidate genes were prioritized for further study in human ERMS based on the following criteria. 1) Candidate oncogenes were differentially expressed in human ERMS compared to ARMS and/or normal muscle as assessed by microarray gene expression studies. 2) Genes that have known oncogenic activity in other cancer types, but yet ascribed functional roles in ERMS, were prioritized for additional study to serve as “proof of concept” genes in our cross-species comparative study. 3) A subset of genes was selected which have unknown function in ERMS and represent potential novel oncogenes. 4) Amplified CNA regions that harbored a single human homologue were also prioritized. Based on these criteria, *CCND2*, *HOXC6*, *PLXNA1*, *VEGF*, *BRAF* and *LIMK1* were selected for further study (Table 1). *CCND2*, *PLXNA1*, *VEGFA*, and *LIMK1* were the single genes contained within the amplified CNA intervals in human disease. *BRAF* was the only gene in the interval that was overexpressed in human ERMS, whereas *CRY1* and *TNNT1* identified within the same amplified interval were not differentially expressed when comparing human ERMS to normal muscle (Fig. S2). *HOXC6* has been reported to be highly expressed in human ERMS compared with ARMS [23], suggesting an possible role in modulating tumor growth. While *CCND2*, a cell cycle regulator, *VEGFA*, an essential regulator of angiogenesis in a variety of cancer types and *BRAF*, an oncogene in a variety of cancers, most likely serve as our “proof of concept” genes for demonstrating functional significance in human ERMS. *LIMK1*, *HOXC6* and *PLXNA1* represent potential novel candidate genes for driving tumor growth of ERMS.

### A subset of amplified genes play essential roles in regulating ERMS proliferation

**Figure 2:**
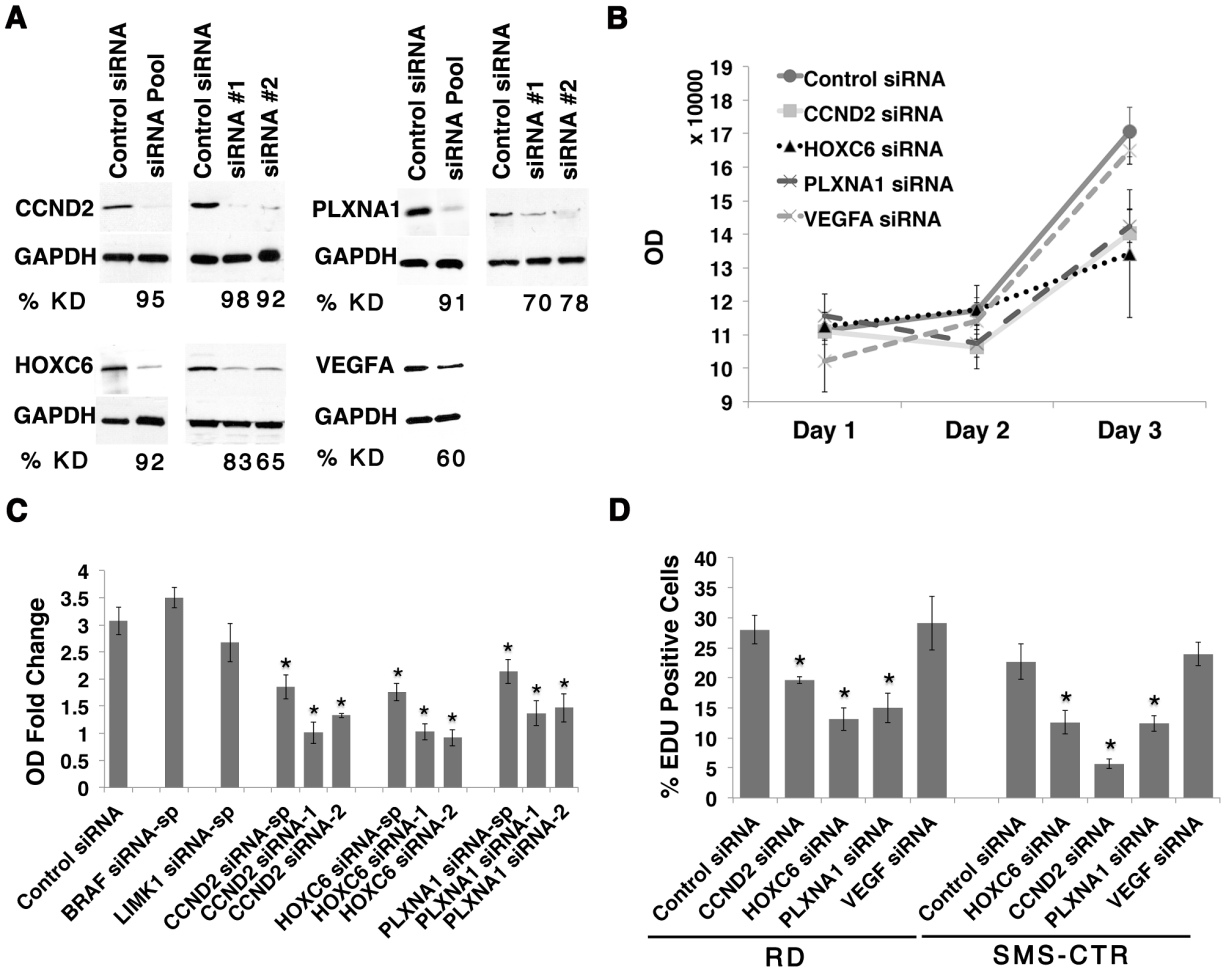
CCND2, HOXC6 and PLXNA1 exert important roles in human ERMS cell proliferation. (A) Western analysis following siRNA knockdown in the RD cell line. The percentage of knockdown (% KD) is indicated below each lane (representative example shown for three independent replicates). (B) Viability following siRNA gene knockdown in RD cells was assessed by cell-titer glo assay. (C) Graph summarizing the results of the cell-titer glo assay. OD-fold change over 3 days in RD cell line from knockdown by smart-pool and two individual gene-specific siRNAs is indicated. (D) Summary of EDU proliferation analysis from RD and SMS-CTR ERMS cell lines,. (Asterisks denote significant differences between siRNA knockdown compared to control siRNA (p<0.05). Each error bar in B, C, and D denotes standard deviation of 3 independent experiments.

The six candidate genes were first assessed for anti-proliferative effects in human RD and SMS-CTR ERMS cell lines by siRNA knockdown, establishing a role for these genes in continued tumor growth and maintenance. Importantly, each of these human ERMS cell lines contains mutationally-activated RAS alleles, mimicking the zebrafish model. Effective gene knockdown was validated by quantitative RT-PCR and/or Western analysis (Fig. 2 A and Fig. S3 B and S4 A). A quantitative VEGFA ELISA confirmed lower levels of secreted VEGFA in the growth medium from cells transfected with *VEGFA* siRNA (Fig. S3 A, p<0.05). Gene-specific siRNA knockdown of *CCND2*, *HOXC6* and *PLXNA1* resulted in reduced cell viability as assessed by a luminescent cell viability assay in both cell lines (Fig. 2 B and S4 B–H). By contrast, knockdown of *BRAF*, *LIMK1*, or *VEGFA* failed to alter viability and/or growth in both cell lines (Fig. 2 B and 2 C and data not shown). Following validation of growth effects using two additional siRNAs for *CCND2*, *HOXC6* and *PLXNA1* in RD and SMS-CTR cell lines (Fig. 2 C and S4 B–H), these genes were prioritized for additional functional studies. For example, knockdown of *CCND2*, *HOXC6* and *PLXNA1* resulted in reduced EDU incorporation when compared to cells transfected with control siRNA in both RD and SMS-CTR cell lines, suggesting that inhibition of cell growth resulted from a block in proliferation (Fig. 2 D). Apoptosis was not altered by gene knock down as assessed by Annexin V staining (Fig. S5). In total, our data uncovered important roles for CCND2, HOXC6 and PLXNA1 in regulating ERMS proliferation, validating the role of several novel genes in regulating continued tumor cell proliferation in human ERMS cells.

### Knockdown of PLXNA1 results in increased terminal differentiation and impaired anchorage-independent growth in human ERMS

**Figure 3:**
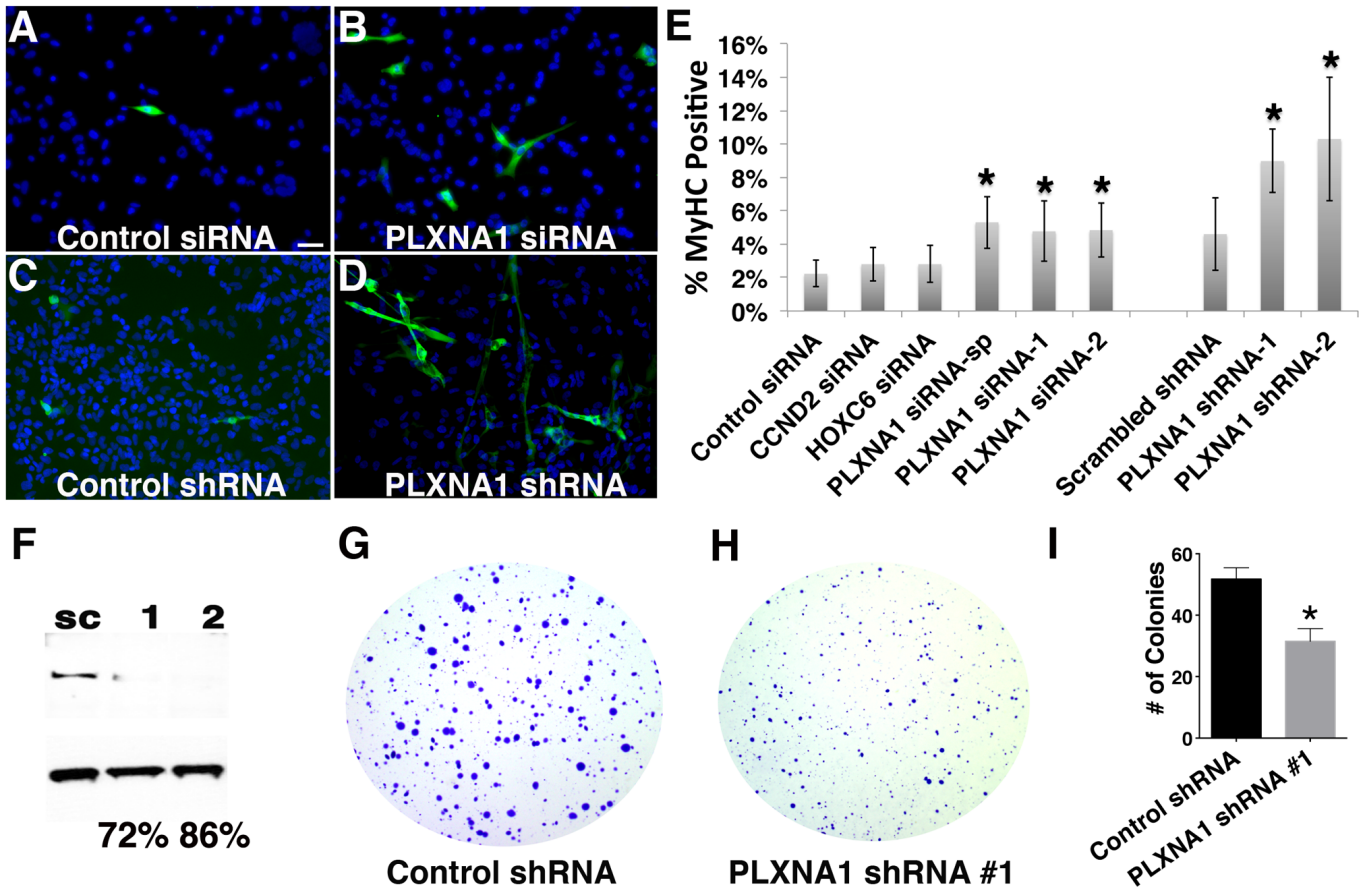
Knockdown of PLXNA1 induced differentiation and impaired anchorage-independent growth of human ERMS cells. RD cells stained with myosin heavy chain (MF20) and DAPI following culture under differentiation conditions for 72 hrs. (A) Control siRNA. (B) *PLXNA1* smart-pool siRNA. (C) Control scrambled shRNA. (D) *PLXNA1* shRNA-1. DAPI, blue; MF20-positive cell, green. (E) Quantification of MF-20 immunofluorescence in siRNA and shRNA-knockdown RD cells. Asterisk indicates significant differences between gene knock- down and control cells (p<0.05). Error bars denote standard deviation. (F) Western analysis of *PLXNA1* shRNA stable knockdown; sc, scrambled control shRNA; 1, *PLXNA1* shRNA-1; 2, *PLXNA1* shRNA-2. A soft agar colony formation assay to assess PLXNA1 knockdown effects on anchorage-independent growth (G–I). (G) Control scrambled shRNA. (H) *PLXNA1* shRNA. (I) Quantification of colony formation assay results. Error bar indicates standard deviation from triplicate experiments.

ERMS expresses myogenic factors such as MYOD and MYF5 yet it fails to complete normal myogenesis secondary to differentiation arrest [24], [25]. As a result, ERMS is composed of heterogeneous subpopulations of proliferating tumor cells that vary in their differentiation status. Therefore, oncogenes that are essential for regulating proliferation of ERMS cells likely also play a role in modulating their differentiation status. Thus, we determined whether CCND2, HOXC6, and PLXNA1 also played a role in blocking differentiation of ERMS. Knockdown of *PLXNA1* resulted in increased formation of multinucleated myocytes and induction of myosin heavy chain expression in RD cells (Fig. 3 B, p = 0.03). By contrast, siRNA knockdown of *CCND2* and *HOXC6* did not alter the differentiation status of human RD cell (Fig. 3 E). To validate the phenotype of *PLXNA1* knockdown, two independent *PLXNA1* shRNA knockdown stable lines were generated and cultured under differentiation condition. Both *PLXNA1* shRNAs induced robust gene knockdown compared to control scrambled shRNA (Fig. 3 F), resulting in increased numbers of multinucleated-myocytes and myosin heavy chain expression (Fig. 3 D–E, p = 0.01).

PLXNA1 also played a critical role in regulating anchorage-independent growth in colony formation assays. Stable knockdown of *PLXNA1* resulted in impaired anchorage-independent growth with decreased colony formation two-fold over 15 days when compared to RD cells transduced with control shRNA (Fig. 3 G–I, p = 0.0003). Moreover, colonies were smaller in size, likely reflecting the prominent role of PLXNA1 in regulating cell growth. Together, our findings indicate that PLXNA1 plays an essential important role in regulating proliferation and differentiation in transformed ERMS.

### Knockdown of PLXNA1 results in impaired migration of human ERMS cell lines

**Figure 4:**
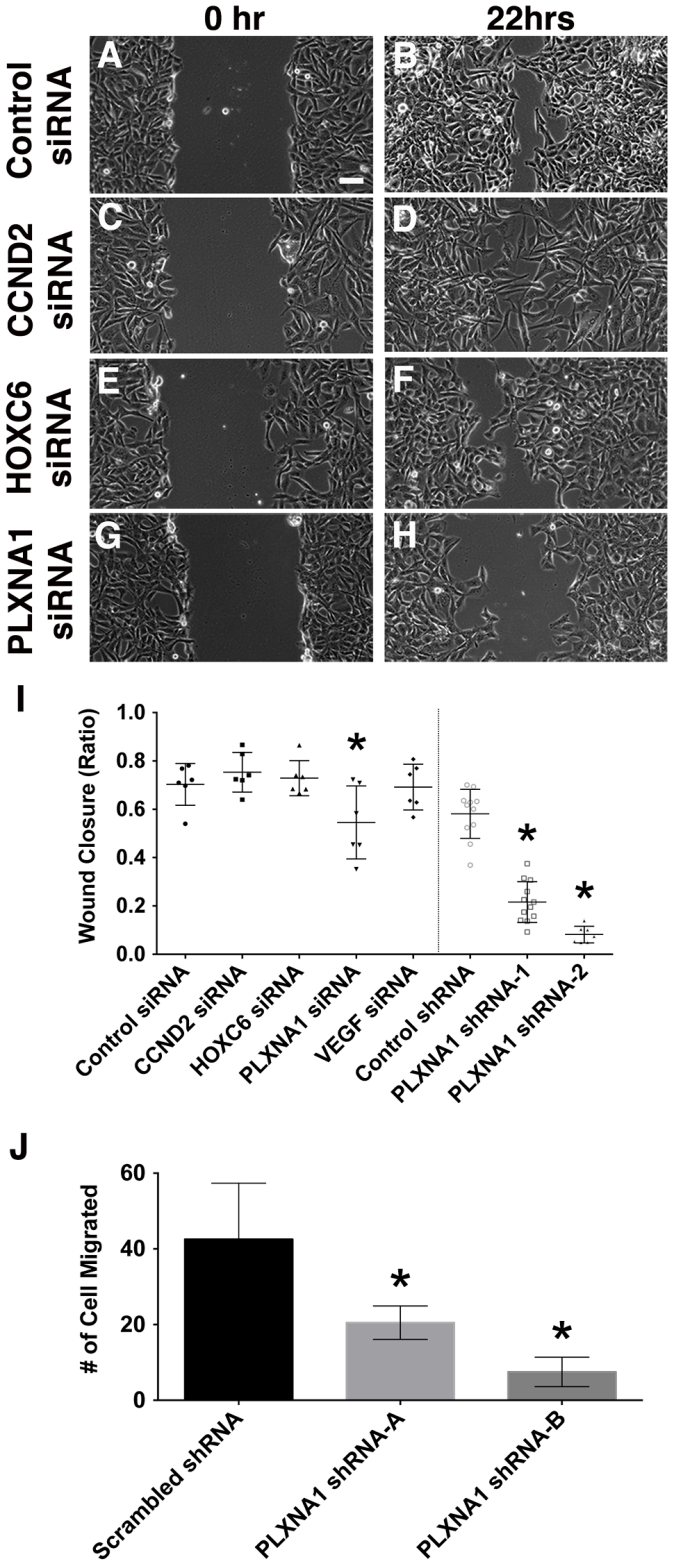
Knockdown of PLXNA1 impairs migration of human ERMS cells. Representative images of ERMS cells transfected with gene-specific siRNAs at 0 hr (A, control siRNA; C, *CCND2* siRNA; E, *HOXC6* siRNA; G, *PLXNA1* siRNA) and 22 hrs (B, control siRNA; D, *CCND2* siRNA; F, *HOXC6* siRNA; H, *PLXNA1* siRNA) following gap creation. Scale bar indicates 100 µm. (I) Quantification of data from wound healing assay. Each error bar indicates standard deviation across 5–6 independent replicates. (J) A Transwell migration assay was performed in RD cells that stably express either a control shRNA or two independent *PLXNA1* shRNAs. Migration was assessed after 24 hours. Each error bar indicates standard deviation across six fields at 200× magnification. Asterisks denote p<0. 05.

Migratory behavior of tumor cells *in vitro* can be a useful predictive index of cell invasion and metastasis *in vivo*. Genes and pathways that are essential for regulating the migratory behavior of tumors cells can likely serve important functions in mediating metastasis and therefore are potential targets for novel therapy. Wound healing and transwell migration assays were used to assess a role for *CCND2*, *HOXC6*, *PLXNA1* and *VEGFA* in migration of human RD and SMS-CTR ERMS cell lines. Knockdown of *PLXNA1* by siRNAs (smart-pool and individual siRNAs) and shRNAs resulted in impaired migration in both RD and SMS-CTR cells over 22 hours (p<0.02 for RD and p≤0.04 SMS-CTR, Fig. 4 G–I, Fig. S6). By contrast, knockdown of *CCND2*, *HOXC6* and *VEGFA* did not affect migration of either RD or SMS-CTR cells (p>0.25, Fig. 4 A–F, I). As an independent assessment of ERMS cell migration, *PLXNA1* stable shRNA knockdown cells were assessed for migration in a transwell assay. Knockdown of *PLXNA1* in RD cells with two independent gene-specific shRNAs resulted in >50% reduction in transwell migration (p = 0.03 for shRNA-A and p = 0.0038 for shRNA-B, Fig. 4 J). Together, these results support an additional role for PLXNA1 in regulating migratory behavior of human ERMS cells.

### Inhibition of VEGFA results in reduced angiogenesis and tumor growth

**Figure 5:**
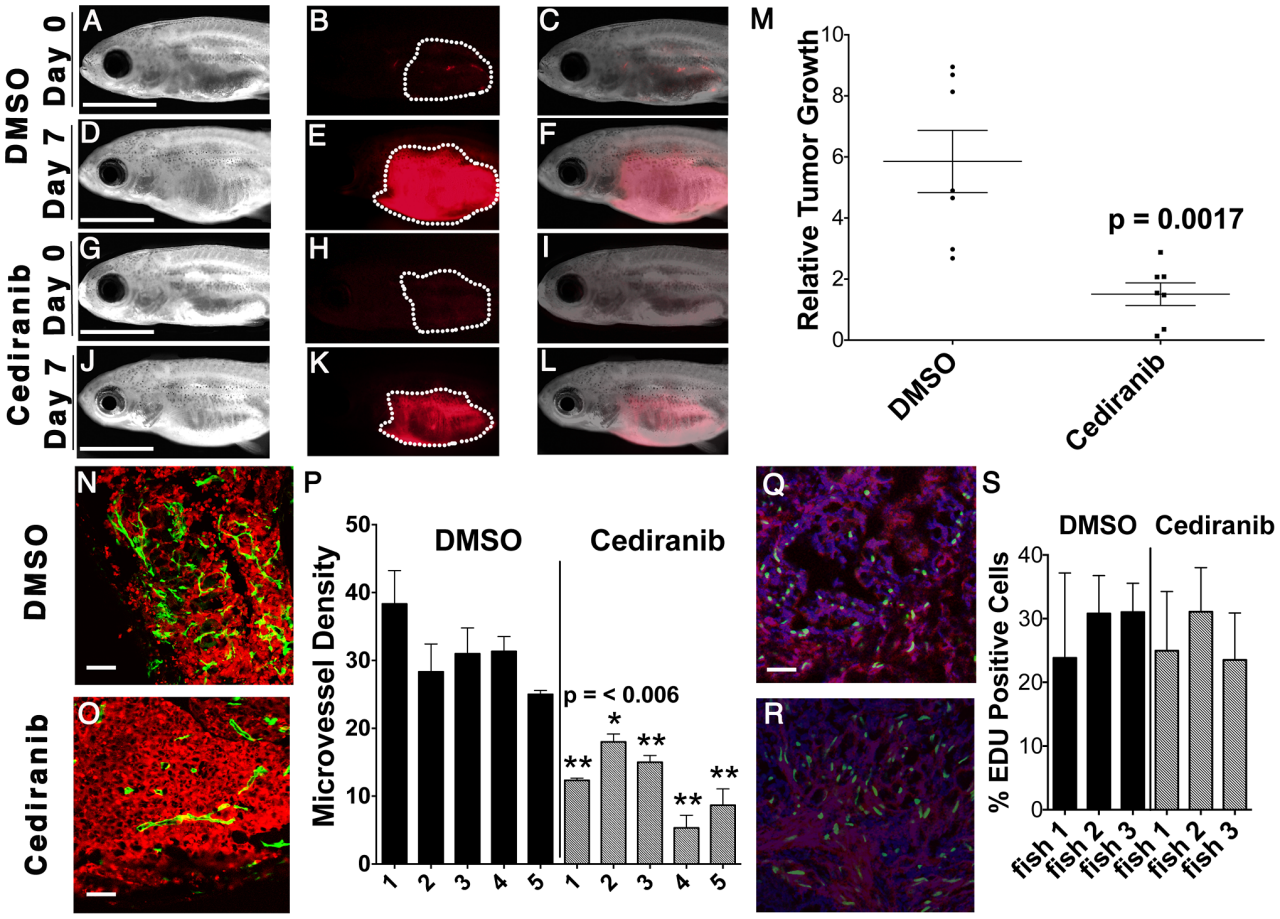
Chemical inhibition of VEGF signaling by cediranib reduces ERMS growth *in vivo*. Syngeneic CG1 fish were transplanted with ERMS cells that co-expressed *rag2-KRASG12D* and *rag2-dsRED*. Fish with engrafted tumors were treated with DMSO vehicle (A–F) or 100 nM of cediranib for 7 days (G–L). Pre-treatment (A–C and G–I) and post-treatment images (D–F and J–L) of representative fish. Bright field (A,D,G,J), dsRED fluorescence (B,E,H,K) and merged image planes (C,F,I,L). Scale bar is 3 mm. (M) Quantification of relative volume change for individual animals. (N–O) *fli1-GFP* transgenic zebrafish were transplanted with dsRED-labeled ERMS and treated with DMSO (N) and cediranib (O). Scale bar equals 50 µm. (P) Microvessel density quantification. Asterisk indicates statistically significant difference between DMSO and cediranib-treated groups based on student t-test. Each error bar indicates standard deviation from 3 fields of microvessels for each animal. EDU incorporation analysis in DMSO (Q) or cediranib (R) treated fish. Scale bar is 50 µm. (S) Quantification of EDU analysis across each cohort of animals. Each error bar indicates standard deviation of percent EDU+ cells found within 3 fields for each animal.

The VEGFA pathway often exerts powerful roles in regulating cancer-induced angiogenesis, which would have been missed in our human cell culture assays. To assess a role for VEGFA in modulating tumor growth *in vivo*, ERMS-bearing zebrafish were treated with the VEGF receptor tyrosine kinase inhibitor, cediranib, or DMSO vehicle for 7 days and assessed for effects on tumor growth. Relative tumor growth as determined by the ratio of tumor volume change between pre- and post-treatment was reduced by three-fold in cediranib-treated fish when compared to those treated with vehicle (Fig. 5A–M, p = 0.0017, Student's T-test). As VEGFA is known to promote angiogenesis during tumor progression in a variety of cancers, we next assessed whether inhibition of VEGFA also blocked angiogenesis in ERMS *in vivo*. In order to visualize angiogenesis in established tumors, ERMS co-expressing *rag2-KRASG12D* and *rag2-dsRED* were transplanted into irradiated *fli1-GFP* fish that exhibit vessel-specific GFP expression [26]. Fish with engrafted ERMS were treated with either cediranib or DMSO vehicle for 7 days. Animals were assessed for differences in both overall tumor growth and microvessel density as determined by cryosections of tumors. ERMS-affected animals treated with cediranib showed a significant reduction in tumor growth with an accompanied two-fold reduction in tumor microvessel density when compared to those treated with vehicle control (N = 5 for each group, p = 0.006, Fig. 5 N–P). Cediranib-treated ERMS did not exhibit a difference in proliferation when compared to vehicle control-treated tumors (Fig. 5 Q–S), consistent with our results for *VEGFA* gene knockdown in human ERMS cell lines. Together, these data suggest that activation of the VEGF pathway promotes ERMS tumor progression through enhanced angiogenesis.

### CCND2, HOXC6, PLXNA1 and VEGFA are commonly expressed in human rhabdomyosaroma

**Figure 6:**
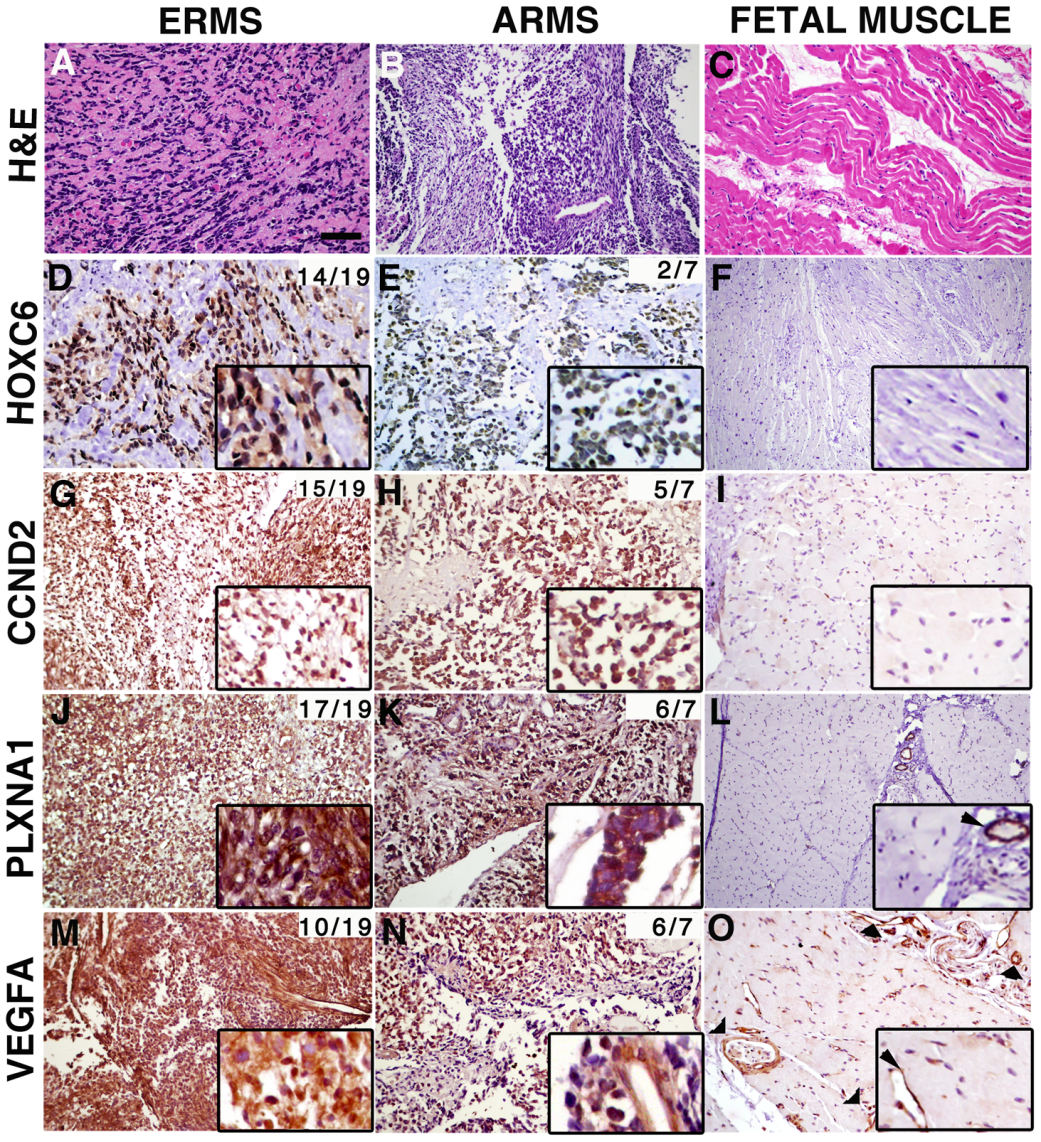
Genes contained within low copy CNAs are expressed in primary human rhabdomyosaroma but not normal fetal muscle. Immunohistochemistry of human primary RMS and fetal muscle tissue samples. Hematoxylin and Eosin stained sections (A–C). Expression of HOXC6, CCND2, PLXNA1 and VEGFA in embryonal rhabdomyosaroma (D, G, J, M), alveolar rhabdomyosaroma (E, H, K, N) and fetal muscle (F, I, L, O). Magnified views of staining are shown in insets. In fetal muscle, PLXNA1 and VEGFA are only expressed in the vasculature (examples indicated by arrowheads in L and O and corresponding insets). The cumulative frequency of positive staining within tumor subtype is shown in the top right corner of each panel (pediatric and adult samples combined). Scale bar (panel A) = 50 µm.

Having established roles for CCND2, HOXC6, PLXNA1 and VEGFA in ERMS growth, we next wanted to assess the extent to which these proteins are expressed in human primary RMS. Immunohistochemistry was performed using antibodies to CCND2, HOXC6, PLXNA1 and VEGFA in primary human tumors and fetal muscle (Supplemental Table S2). In all, 8 pediatric and 11 adult ERMS and 3 pediatric and 4 adult alveolar RMS (ARMS) were analyzed. Remarkably, CCND2, HOXC6, PLXNA1 and VEGFA protein expression were detected in a majority of RMS samples while antibody staining for each was largely negative in fetal muscle (Fig. 6). Specifically, HOXC6 protein expression was detected in 14 of 19 ERMS with strong, diffuse staining being found in 6 of the 14 cases (1 adult and 5 pediatric). In contrast, only 2 of 7 ARMS showed weak, positive staining for HOXC6, consistent with lower-level gene transcript levels being detected in pediatric ARMS compared to ERMS (Supplemental Fig. S7). CCND2, PLXNA and VEGFA were expressed at comparable frequency in both subtypes of RMS. For example, CCND2 was detected in 15 of 19 ERMS and 5 of 7 ARMS, while PLXNA1 expression was found in 17 of 19 ERMS and 6 of 7 ARMS. VEGFA antibody staining was detected in the tumor cells and the vasculature in 10 of 19 ERMS (strong staining in 1 adult and 1 pediatric case), while 6 of 7 ARMS exhibited weak staining in all cases analyzed. Additional immunohistochemical analysis of a tissue microarray from Children's Oncology Group revealed positive VEGF expression in 31 of 38 ERMS and 3 of 6 ARMS (Table S3). Of the 38 cases of ERMS, 29 cases showed strong and diffuse staining. Our analysis suggests that despite these oncogenes being infrequently amplified in human disease, their protein expression levels are elevated in a majority of human ERMS. These data imply important roles for these genes in regulating tumor growth in a large fraction of human ERMS and suggesting additional, as of yet undiscovered mechanisms that regulate expression of these genes.

### High VEGFA expression correlates with clinical outcome

**Figure 7:**
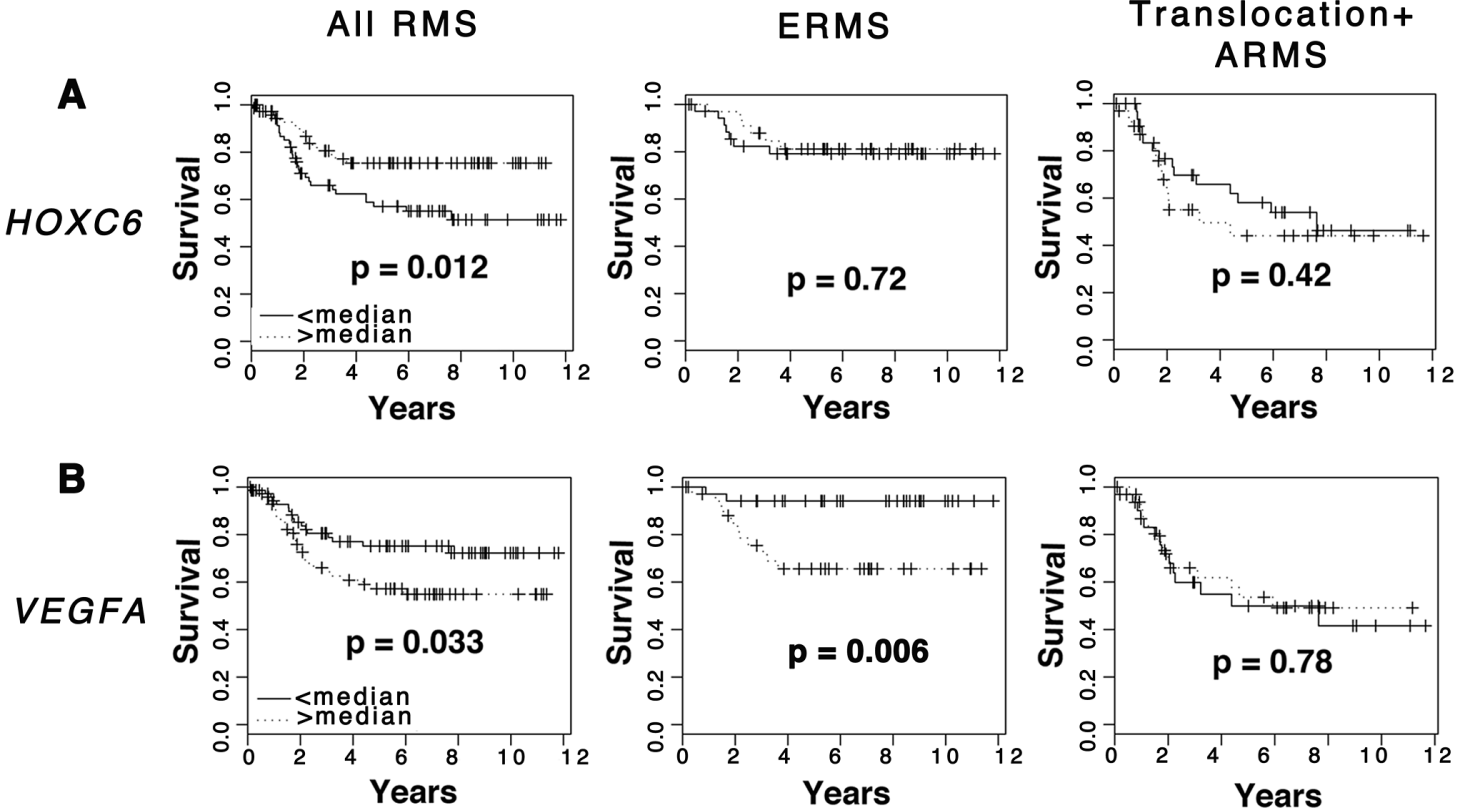
Elevated *VEGFA* expression correlates with poor clinical outcome in rhabdomyosarcoma. Kaplan-Meier analysis was completed using microarray gene expression data for which patient outcome is available. Comparison was made in all RMS patients, ERMS patients only, and translocation-positive ARMS patients only. (A) *HOXC6*. (B) *VEGFA*.

To assess whether dysregulated expression of *CCND2*, *HOXC6*, *PLXNA1* and *VEGFA* correlates with clinical outcome, Kaplan Meier analyses were completed using microarray gene expression data from primary ERMS and ARMS [23]. Samples were stratified based on high and low median expression for each gene and each assessed as an independent predictor of survival. Based on this analysis, differential expression of *CCND2* and *PLXNA1* did not correlate with overall survival outcome in either ERMS or ARMS (Fig. S8). *HOXC6* was differentially upregulated in ERMS compared to ARMS (Fig. S7); thus, high expression of *HOXC6* correlated with better overall survival (Fig. 7 A), a finding consistent with previous studies demonstrating better clinical outcome for ERMS patients compared to those with ARMS [27]. Finally, samples with high mRNA expression of *VEGFA* correlated with low overall clinical survival in the ERMS cohort but did not predict survival outcome in ARMS (Fig. 7 B). In addition, *VEGFA* expression did not correlate with clinical stage, indicating that it is likely an independent prognostic indicator (Fig. S9). These data implicate important roles of VEGFA in promoting ERMS tumor progression and identify VEGFA as a biomarker with likely use in stratifying ERMS patients into high and low-risk groups.

## Discussion

Prior cytogenetic and array CGH studies in human ERMS demonstrate inconsistent and non-specific partial to whole chromosomal aneuploidy across different primary tumors making it difficult to identify critical genes essential for driving tumor growth. Utilizing a zebrafish model of RAS-induced ERMS that mimics the human disease [4], [21] and subsequent array CGH analyses of genomic DNA from tumor vs. matched normal, we were able to rapidly identify candidate gene-containing regions that likely contribute to ERMS pathogenesis. The 19 CNA gains that were recurrently amplified in zebrafish ERMS mapped to 21 homologous regions within the human genome. Remarkably, 18 of these regions also demonstrated low-level genomic amplification in human ERMS. To validate that candidate genes contained within these intervals exert important roles in continued tumor growth and maintenance, we characterized the function of six amplified genes in human ERMS cell lines and conclusively demonstrated functional significance of CCND2, HOXC6, PLXNA1 in proliferation of human ERMS. PLXNA1 also has important roles in regulation differentiation and migration of ERMS cells. As the *in vitro* analyses performed in this study would not be able to assess other aspects of tumorigenesis such as neovascularization and tumor initiation, we utilized the zebrafish *in vivo* model to demonstrate the important role of VEGF-A pathway in mediating angiogenesis during tumor growth. In total, our work identified roles for 4 of 6 candidate genes identified in our cross-species array CGH studies for eliciting important roles in human ERMS. Importantly, this strategy is not limited to zebrafish ERMS, and will likely provide powerful new methods to identify novel tumor-suppressor and oncogenes in a wide range of zebrafish and human tumors.

Data from our array CGH study and previous studies of zebrafish cancer revealed low-level CNA gains as a frequent DNA alteration in cancer, yet this class of mutation has not commonly been studied due to the difficulty in identifying relevant and meaningful genes in these regions. Importantly, zebrafish allows for the easy identification of low-level gene amplifications. In total, our data is consistent with a model where zebrafish tumor cells undergo acquisition of low-amplitude gains, likely represented as single copy gains within CNA regions. For example, we have also observed that clonal-populations of purified T-ALL cells (90% enriched for blasts) also contain low-amplitude gains [Blackburn et al., unpublished]. Moreover, Rudner et al. (2011) recently showed that a majority of amplified, gene-containing CNAs found in zebrafish T-ALL were also amplified in human disease [20]. Upon re-analysis of this data, we find that 72% of the reported amplified regions were detected as low-level gains in zebrafish T-ALL, yet were not reported as such. Zhang et al. identified large regions of aneuploidy and high-level CNA gains in zebrafish malignant peripheral nerve sheath tumors when assessed by array CGH, but also identified numerous regions of low-level CNA gains, which were dismissed as potential causative lesions in cancer. Thus, despite these previous two reports observing low-level CNA gains in zebrafish malignancy, neither reported the functional importance of this class of genes to promote tumor progression and maintenance in zebrafish or human disease. Although it is formally possible that low level gains detected in zebrafish ERMS represent high-copy gains masked by a high degree of tumor cell heterogeneity and/or contamination of normal DNA from non-transformed blood, fibroblasts and stroma, our data strongly argue that low-copy amplification is a common attribute found in zebrafish and human cancer.

Interestingly, even though the functional relevance of low-level gains such as genomic duplication events have been infrequently reported in human cancer, this type of DNA alteration often predicts important clinical parameters such as disease susceptibility, therapy resistance and adverse prognosis. For example, duplication of a region on chromosome 6q27 is detected in individuals affected with familial chordoma, a rare bone cancer, but not among unaffected individuals within the same family [28]. *MYB* tandem duplication occurs in pediatric T-ALL and results from homologous recombination at ALU repetitive sequences flanking the *MYB* locus. Elevated *MYB* expression is associated with poor outcome in T-ALL [29]. Similarly, focal tandem duplication also contributes to chemotherapy resistance in patients with high-grade ovarian cancer [30]. These findings indicate that low-level CNA gains have important clinical prognostic relevance and likely play important functional roles in human cancer. Finally, we also found that genes within each CNA are highly expressed in a majority of human RMS despite being infrequently amplified as low-copy CNAs, suggesting the importance of these gene pathways in regulating a large fraction of human ERMS and that additional mechanisms underlying the dysregulation of this class of genes in cancer is likely.

Our work has identified essential roles for four genes in modulating ERMS growth, maintenance, migration, and neovascularization. Of these genes, CCND2, HOXC6 and PLXNA1 exhibited important roles in regulating proliferation in human ERMS cell lines. PLXNA1 also had additional roles in arresting ERMS cells in early stages of muscle differentiation, in enhancing tumor cell migration, and in altering anchorage-independent growth. Despite the fact that these genes and/or related family members have been ascribed functions in other cancer types, their contributions to the pathogenesis of ERMS have not been previously characterized. For example, HOXC6, a homeobox transcription factor, regulates the expression of genes including BMP7, FGFR2, IGFP3 and PDGFRA to influence oncogenic activities in prostate cancer [31]. HOXC6 is highly expressed in ERMS but not ARMS [23], suggesting a specific and independent role in regulating growth in the human ERMS subtype. A role for HOXC6 in regulating continued RMS growth had not been reported until this study. CCND2 belongs to the D-type G1 cyclins (D1, D2 and D3). While cyclin D1 is frequently dysregulated in cancer and is a marker for disease progression [32], the involvement of cyclin D2 in cancer is not as well characterized. CCND2 is amplified in 2% of gliomas and in zebrafish and human MPNSTs [19], [33]. Finally, PLXNA1 belongs to a highly conserved family of transmembrane receptors that bind semaphorins and have been shown to mediate neuronal cell migration, guidance, and patterning [34], [35]. In humans, nine plexins group into four subfamilies and several have been implicated as having roles in cancer progression and growth. In particular, plexin-B1 can function as an oncogene by promoting proliferation and survival of B-Cell Lymphoblastic Lymphoma cells and invasion of ovarian and breast tumor cells [36]–[38]. Plexin-A1, the gene identified in our study, has been shown to activate the VEGF receptor and NF-κB to promote survival of malignant mesothelioma cells [39], suggesting a complex interplay of PLXNA1 in cell survival and neovascularization. Taken together, our study has demonstrated prominent and novel roles for CCDN2, HOXC6, and PLXNA1 in modulating ERMS proliferation while PLXNA1 exerts important additional roles in regulating differentiation and migration. None of these genes have been previously implicated as important modulators of ERMS growth and maintenance, suggesting that our cross species array CGH studies will be valuable for uncovering genetic lesions across a wide range of zebrafish and human cancers.

VEGF pathway activation promotes tumor angiogenesis and progression in a variety of human cancers, and elevated VEGF expression correlates with poor prognosis in certain tumor types [40]–[42]. However, until our report, the prognostic impact of *VEGFA* expression in human ERMS had not been described. Here, we show that *VEGFA* is amplified as a low-copy gain in a small cohort of zebrafish and human RMS and yet highly expressed in a majority of human patient samples. High *VEGFA* mRNA expression correlated with poor clinical outcome in human ERMS, underscoring the importance of this pathway in driving continued tumor growth. As *VEGFA* expression level is not linked to clinical stage, it represents an important independent prognostic indicator and a potential biomarker for therapy stratification. Chemical inhibition of VEGF signaling in our pre-clinical *in vivo* model effectively suppressed tumor growth by reducing angiogenesis, consistent with the findings from a pre-clinical testing of VEGFR inhibitors on a small number of human RMS xenografts into mice [43]. Although clinical trials of VEGF inhibitors in other types of cancers have exhibited mixed results [44]–[46], our data suggest that targeting the VEGF pathway may be a promising therapeutic option to curb tumor growth in a subset of high-risk ERMS patients.

In summary, our array CGH studies of zebrafish cancer have identified conserved CNA gains with functional significance in human ERMS. As proof of principle, we have also demonstrated the utility of zebrafish array CGH studies to identify oncogenes that are essential for continued tumor growth in both zebrafish and human ERMS. Our work also provides 13 additional CNA gains that are conserved in zebrafish and human ERMS for which an essential genetic lesion has yet to be identified – providing potential genes to interrogate in the future. Our studies suggest that most amplified CNAs will contain genes that regulate important processes in cancer maintenance and growth. Moreover, our study reveals a number of tractable features of zebrafish cancer genomes such as small-size CNAs containing few genes within each region of chromosomal aberration, thereby positioning the zebrafish as an effective model system for discovering novel genes required for continued tumor growth and maintenance within a wide diversity of cancer types.

## Methods

### Animal and human protocol approval

Studies were approved by the Massachusetts General Hospital Subcommittee on Research Animal Care under protocol #2011N000127 (zebrafish) and by the Partners Human Research Committee under IRB protocol #2009-P-002756 (human).

### Array comparative hybridization

TuAB-strain zebrafish were co-injected at the one-cell stage with linearized *rag2-KRASG12D* and *rag2-dsRED* DNA constructs as previously described [4], [47]. dsRED-labeled ERMS and adjacent non-neoplastic tissues were dissected from tumor-bearing animals at 30–40 days of life. RNA and DNA were extracted by Trizol (Sigma). Tumor DNA was labeled with Cy5 (Bioprime system, Invitrogen, Carlsbad, CA) and hybridized against the matched normal samples labeled with Cy3 onto the custom SurePrint G3 400k CGH microarray (Agilent Technologies, Santa Clara, CA). Array image scans were extracted using Agilent Feature Extraction software (Agilent Technologies, Inc, Santa Clara, CA), normalized for signal intensity, and imported into the Nexus Copy Number software program version 5.1 (Biodiscovery, Inc., El Segundo, CA). CNA calls were generated based on log_2_ ratio output files using a rank segmentation algorithm. Settings were optimized using self-self hybridizations to reduce false positive calls. The parameters include significance threshold 1.0 E-5, maximum continuous probe spacing of 200 kb, minimum number of probes per sequence of 3, and log_2_ ratios of 1.0, 0.25, −0.25 and −1.0 for high-level amplifications, gains, losses and deletions, respectively. CNAs of interest were determined using the aggregate function in Nexus. Aggregates are represented as segmented regions of gain or loss shared by a set of samples with the number of samples sharing the event referred to as the aberration frequency. The minimum aberration frequency required for analysis in our study was set at ≥15% (n≥3 of 20 zebrafish ERMS contained a common region of gene amplification).

For the human ERMS sample analysis, normalized log_2_ intensity files (series number GSE27392) were downloaded from Gene Expression Omnibus (GEO) at the National Center for Biotechnology Information (NCBI) and imported into and analyzed using Nexus Copy Number software (version 5.1, BioDiscovery). This program analyzes log_2_ ratio output files using a rank segmentation algorithm similar to circular binary segmentation. Samples were segmented following the removal of the greatest 3% of outliers and a minimum five-probe requirement at a significance threshold of 1E-08. Gains and losses were defined as regions exhibiting log_2_ values of 0.2 and −0.18, respectively, with high-level amplifications and deletions defined as log_2_ values greater than 0.5 and less than −0.5. Following the identification of human ERMS aberrations, homologous human regions of zebrafish ERMS CNAs were analyzed to determine whether common low-level amplifications were present in both zebrafish and human ERMS samples.

### Immunohistochemistry

Paraffinized human primary rhabdomyosaroma (5 ERMS and 3 ARMS), US Biomax tissue microarray (14 ERMS and 4 ARMS, Rockville, MD), and a Children's Oncology Group tissue microarray were analyzed by immunohistochemical staining as previously described [48]. HOXC6 (Sigma, 1∶200), CCND2 (Ab-Cam, 1∶100), PLXNA1 (Ab-Cam; 1∶200) and VEGFA (Ab-Cam; 1∶125). BGAR- biotinylated goat anti-rabbit (Vector #BA-1000) was used as the secondary antibody. Pathology review was completed independently by E.C. and G.P.N.

### Cell lines, siRNA transfection, stable shRNA knockdown and Western analysis

The human RD cell line was obtained from ATCC cell biology collection (Manassas, Virginia) and the SMS-CTR cell line provided by Dr. Corrine Linardic (Duke University, North Carolina). Cells were seeded at a density of 5×10^2^ cells in 6-well plates in 2 ml of antibiotic-free 10% FBS/DMEM. 50 pg of gene-specific smart-pool or control siRNA were transfected into cells using RNAiMax lipofectamine transfection reagent (Invitrogen). For stable knockdowns, scrambled and gene-specific shRNAs in pLKO.1-based lentiviral vectors were packaged in 293T cells. shRNAs were obtained from molecular profiling laboratory at the Cancer Center of Massachusetts General Hospital (Table S4). RD cells were infected with viral particles for 24 hours at 37 degrees with polybrene (Millipore) at 4 µg/mL and then selected with puromycin (In Vivo Gene) at 10 µg/mL in 10%FBS/DMEM for 15 days to obtain stable lines. Total cell lysates from knockdown experiments were immunoblotted using primary antibodies against HOXC6 (1∶500), CCND2 (1∶1000), PLXNA1 (1∶1000) and VEGFA (1∶1000). All Western analysis was completed three times per experiment and average percent knockdown is noted. Incubation with HRP-conjugated secondary antibody (1∶2000) was performed in 5% milk/TBST for 2 hours.

### Cell proliferation and apoptosis assays

siRNA transfected cells were assessed by Cell Titer Glo assay as per the manufacturer's instructions (Promega). Cells were also pulsed with EDU for 2 hours, harvested at 48 hours post-transfection and processed using the EDU ClickIt Flow Cytometry Assay kit (Alexa Fluor 647 dye, Invitrogen). Unstained cells were used as the negative sample to facilitate gating in flow cytometry. To assess apoptosis, cells were harvested at 48 hours post-transfection and labeled with PE Annexin V and 7-AAD using the PE Annexin V Apoptosis Detection Kit (BD Pharmagin). Unstained cells, cells treated with PE Annexin V only and 7-AAD only were used to set up gates for flow cytometry. Each analysis was performed in triplicate. A student's T-test was performed to assess whether the difference in the percentage of Annexin V-positive cells between test samples and control siRNA-transfected cells was significant.

### Cell migration assays

A wound-healing assay was performed in cells transiently transfected with siRNA and/or cells that stably express a gene-specific shRNA. Cell were seeded into 6-well plates and grown to nearly confluent density. A scrape was made in each well using a pipette tip, and cell migration across the gap was assessed after 22 hours. Images were taken at 0 and 22 hrs to calculate the percentage of gap closure. ERMS cells were also analyzed for altered migration in a transwell assay. Specifically, 2×10^4^ cells were seeded in 6.5 mm-membrane inserts (Corning) in DMEM and were allowed to migrate through the permeable membranes (8.0 µM pore size) toward the bottom chamber containing medium with 10% FBS. Cells were then fixed with 4% paraformaldehyde after 24 hours and stained with hematoxylin for 30 minutes. Unmigrated cells from the inserts were removed. Six random fields of the migrated cells on the membranes were imaged using the Olympus light microscope (Model MVX10, 400× magnification) and manually counted. A Student's T-test was performed to assess differences between the control and experimental groups.

### Soft-agar colony formation assay

A base layer of 1% agar in 10% FBS/DMEM was prepared in 6-well plates. Cells were resuspended in 0.5% low-melting point agarose/10%FBS/DMEM and overlaid on the base layer with 2.5×10^3^ cells per well and subsequently kept in the humidified incubator with 5% CO_2_ with media change every 3 days for 15 days. Cells were fixed with 4% paraformaldehyde and stained with 0.05% crystal violet. Colony formation assay for each shRNA stable knockdown line was performed in triplicate. Image for each well containing soft agar colonies was taken at low magnification by light microscopy. Colony count was performed using the ImageJ software and differences assessed by Student T-test.

### Chemical treatment of zebrafish with ERMS

Six-week old CG1 syngeneic fish were transplanted with 3×10^4^ unsorted tumor cells arising from dsRED-positive ERMS from CG1 strain fish (Mizgireuv and Revskoy 2006; Smith et al., 2010). Engrafted animals were treated at 6-days post-transplantation with 100 nM of cediranib (Selleck) and vehicle control (DMSO) for 7 days (including 2 24-hr drug holidays). Tumor volume was assessed by imaging animals pre-treatment and post-treatment. Tumor volume was calculated by multiplying tumor area by fluorescent intensity using image J. A Student's T-test was performed to assess differences between tumor size in the control and experimental groups.

### Estimating microvessel density

Six-week old *fli1-GFP* fish were irradiated at 25 Gy and transplanted with 3×10^4^ unsorted ERMS cells from fish with dsRED-positive ERMS. Fish with engrafted tumors were treated with cediranib as described above. At the end of treatment period, tumor tissues were isolated, fixed in 4% paraformaldehyde for 30 minutes and snap frozen. 5 µM Frozen sections were mounted in DAPI-containing Vectashield (Invitrogen). GFP and dsRED images were obtained at 200× magnification using a Nikon confocal microscope. Microvessel density was quantified using Weidner et al. criteria [49] and differences assessed by Student T-test.

### Kaplan-Meier analysis

Kaplan-Meier analysis was completed using R with the survival package. Median expression level for each gene was used to group samples into high and low expression. Chi-squared tests were used to assess overall survival differences between groups. Statistical significance was defined as a p-value less than 0.05.

## Supporting Information

Figure S1 Validation of copy number changes within CNAs identified by array CGH in zebrafish ERMS. A. Quantitative PCR was performed on genomic DNA extracted from representative tumor/matched normal tissues. Each tumor sample was normalized to matched normal tissue. Each error bar indicates standard deviation from triplicate experiments. B. Corresponding array CGH analysis showing CNA calls based on a log2 scale.
(PDF)

Figure S2 *BRAF* is the only gene within this CNA interval that is upregulated in human ERMS compared to normal muscle. Wisker plots showing relative mRNA expression levels of *BRAF* (A), *CRY1* (B) and *TTNT1* (C) in human ERMS and ARMS with *PAX3-FKHR* fusion and *PAX7-FKHR* fusion in comparison with juvenile muscle as assessed by microarray gene expression.
(PDF)

Figure S3 Knockdown of candidate genes and inhibition of VEGFA activity in ERMS cell lines. (A) ELISA to assess VEGFA protein level in growth media of transfected cells. (B) qRT-PCR to assess knockdown of mRNA levels of candidate genes. (Error bars indicate standard deviation of each triplicate within each experiment). Asterisks denote significant differences with p<0.003 and p<0.008, respectively within each comparison.
(PDF)

Figure S4 Knockdown of CCND2, HOXC6 and PLXNA1 results in reduced growth of human SMS-CTR and RD cell lines. (A) Western analysis of CCND2, HOXC6 and PLXNA1 knockdowns in SMS-CTR cell line using smart-pool (sp) and two individual gene-specific siRNAs. Bottom row: GAPDH. Percentage knockdown for each siRNA is as follows: *CCND2* siRNA-sp, 98%; *CCND2* siRNA#1, 57%; *CCND2* siRNA#2, 91%; *HOXC6* siRNA-sp, 82%; *HOXC6* siRNA#1, 88%; *HOXC6* siRNA#2, 75%; *PLXNA1* siRNA-sp, 77%; *PLXNA1* siRNA#1, 94%; *PLXNA1* siRNA#2, 54%; *VEGFA* siRNA, 97%. (B) Summary of cell-titer glo analysis in SMS-CTR cell line. OD fold changes over 3 days for smart pool (sp) and individual siRNAs are shown. Asterisk indicates statistical significance in comparison to control siRNA treatment by Student's t-test (p<0.05). Cell-titer glo assay showing OD change in 3 days for RD (C–E) and SMS-CTR (F–H) cells transfected with two individual gene-specific siRNAs. Each error bar indicates standard deviation of triplicate experiments.
(PDF)

Figure S5 Knockdown of CCND2, HOXC6, PLXNA1 and VEGFA did not affect apoptosis. Annexin V analysis was performed on RD and SMS-CTR cells transfected with siRNA targeted against CCND2, HOXC6, PLXNA1 and VEGFA. Each error bar indicates standard deviation of triplicate experiments.
(PDF)

Figure S6 Knockdown of PLXNA1 results in impaired migration in a wound-healing assay. Cells transfected with siRNA or shRNA were allowed to migrate over a scratch wound over 22 hours. Representative images of cells with control siRNA, two gene-specific *PLXNA1* siRNAs, control shRNA and two gene-specific shRNAs at 0 hr (A–F) and 22 hrs (G–L) are shown. Scale bar indicates 50 µm. (M) Summary of assessing PLXNA1 knockdown in wound healing assays using two gene-specific siRNAs in RD and SMS-CTR cell lines. Eight random measurements at each time point were made for each siRNA. The ratio of wound closure was determined by the difference in distance migrated over total distance. Each error bar denotes standard deviation. (N) Summary of wound healing assays using two gene-specific *PLXNA1* shRNAs.
(PDF)

Figure S7 HoxC6 is differentially expressed in ERMS when compared to ARMS and normal muscle. Wisker plot showing relative mRNA expression levels of *HOXC6* in human RMS in comparison with juvenile and adult muscle as assessed by microarray gene expression. Asterisk denotes statistical significance based on Student's t-test (p<0.001). PAX3-FKHR+ ARMS (P3+ ARMS), PAX7-FKHR+ ARMS (P7+ ARMS).
(PDF)

Figure S8 Kaplan-Meier analysis correlating expression levels of *CCND2* and *PLXNA1* with clinical survival. Kaplan-Meier analysis was completed using microarray data from Davicioni et al (2010) to correlate expression levels of *CCND2* and *PLXNA1* with clinical survival. Comparison was made in all RMS patients, ERMS patients only, ARMS patients only and non-RMS patients. (A) *CCND2*. (B) *PLXNA1*.
(PDF)

Figure S9 *VEGFA* transcript expression does not correlate with clinical stage of ERMS. Kaplan-Meier analysis was completed using microarray data from Davicioni et al (2010). (A) Stage 1. (B) Stage 2. (C) Stage 3. (D) Stage 4. Normalized mRNA expression levels of VEGFA across all RMS (E), ERMS (F) and ARMS (G) samples were also assessed in box plots.
(PDF)

Table S1 Summary of zebrafish samples with recurrent regional gains and deletions in array CGH analysis.
(PDF)

Table S2 Summary of clinical information and immunohistochemical staining results of human RMS samples.
(PDF)

Table S3 Summary of clinical information and immunohistochemical staining results of RMS samples in the Children's Oncology Group tissue microarray.
(PDF)

Table S4 Summary of shRNA sequences and quantitative primers used in array CGH validation studies.
(PDF)
