# Supplementary figures and images for: Cross-Species Array Comparative Genomic Hybridization Identifies Novel Oncogenic Events in Zebrafish and Human Embryonal Rhabdomyosarcoma
Source: PLoS Genet. 2013 Aug 29;9(8):e1003727. doi: 10.1371/journal.pgen.1003727 (PMC3757044; doi:10.1371/journal.pgen.1003727)

**Fig. S1**

**A**

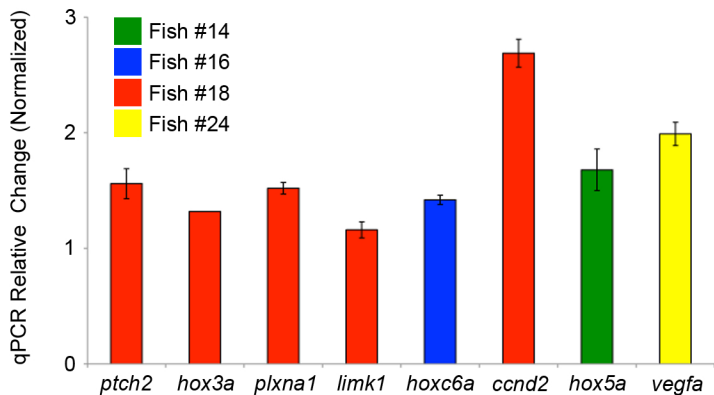

**B**

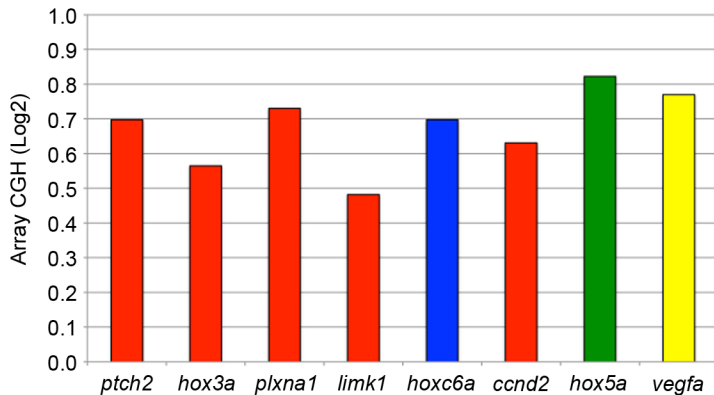

Supplement: Figure S1 — Validation of copy number changes within CNAs identified by array CGH in zebrafish ERMS. A. Quantitative PCR was performed on genomic DNA extracted from representative tumor/matched normal tissues. Each tumor sample was normalized to matched normal tissue. Each error bar indicates standard deviation from triplicate experiments. B. Corresponding array CGH analysis showing CNA calls based on a log2 scale. (PDF) [file pgen.1003727.s001.pdf]

# Fig. S2

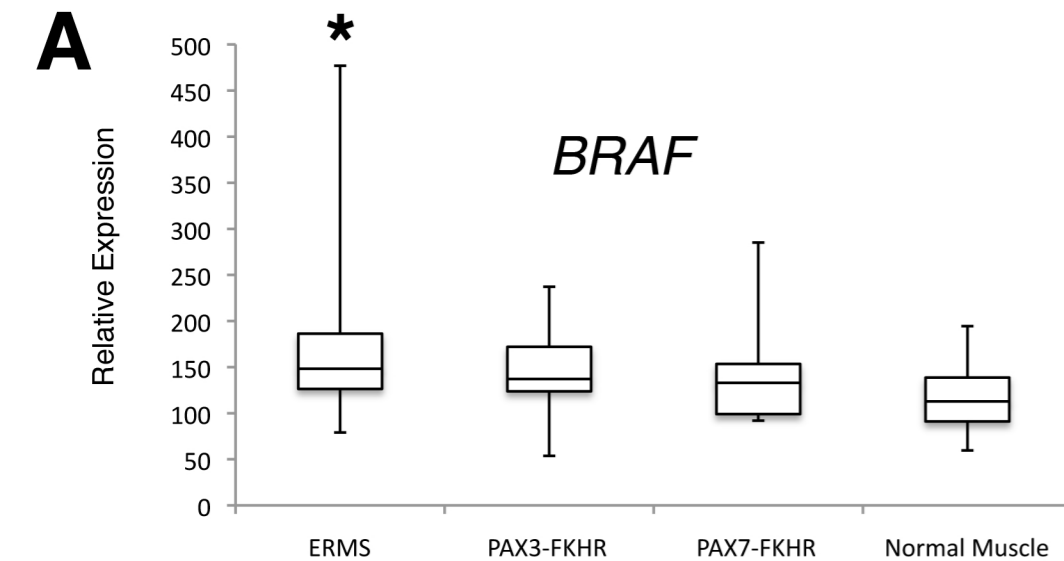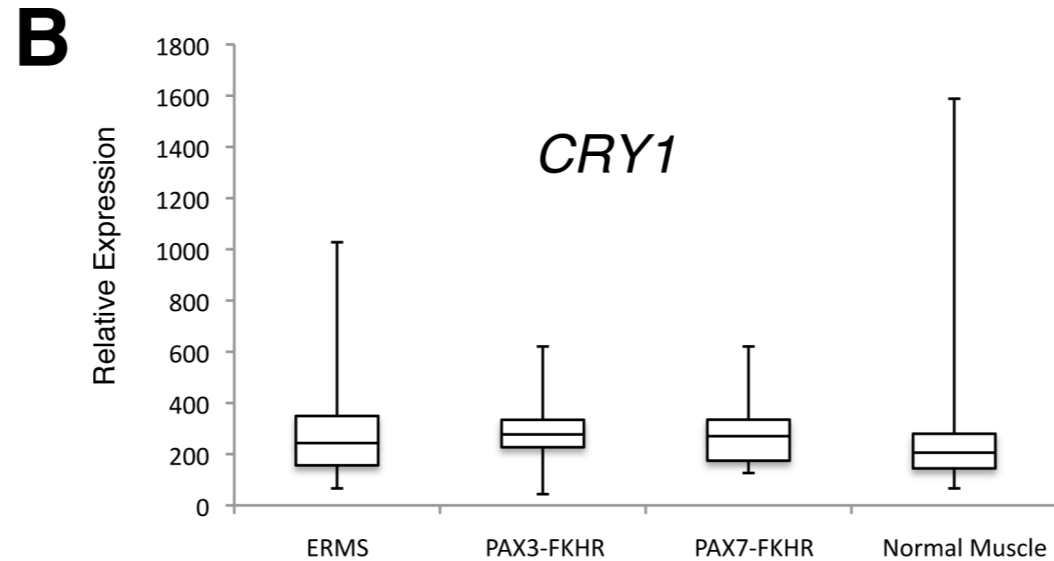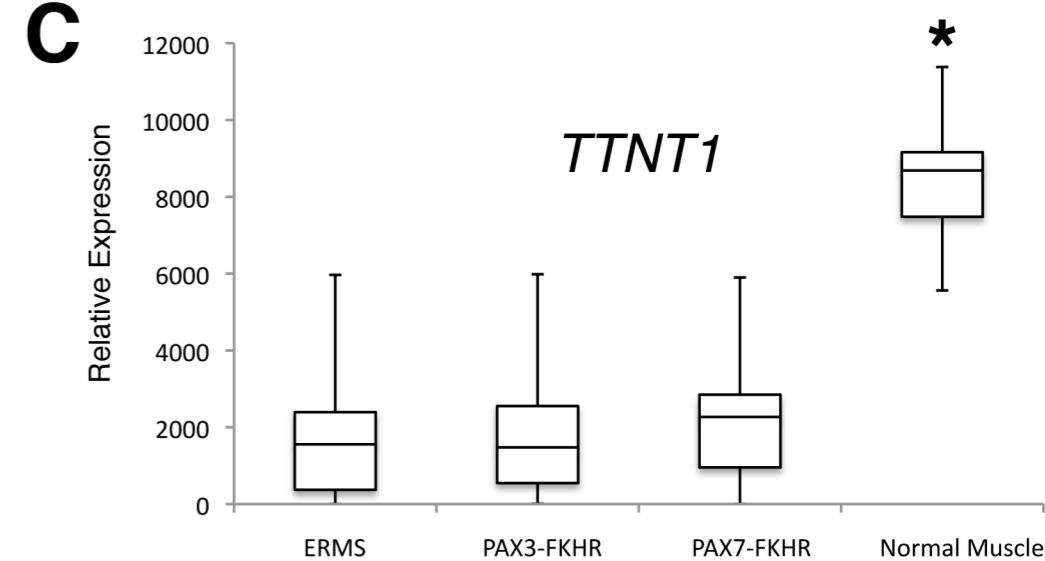

Supplement: Figure S2 — BRAF is the only gene within this CNA interval that is upregulated in human ERMS compared to normal muscle. Wisker plots showing relative mRNA expression levels of BRAF (A), CRY1 (B) and TTNT1 (C) in human ERMS and ARMS with PAX3-FKHR fusion and PAX7-FKHR fusion in comparison with juvenile muscle as assessed by microarray gene expression. (PDF) [file pgen.1003727.s002.pdf]

**Fig. S4**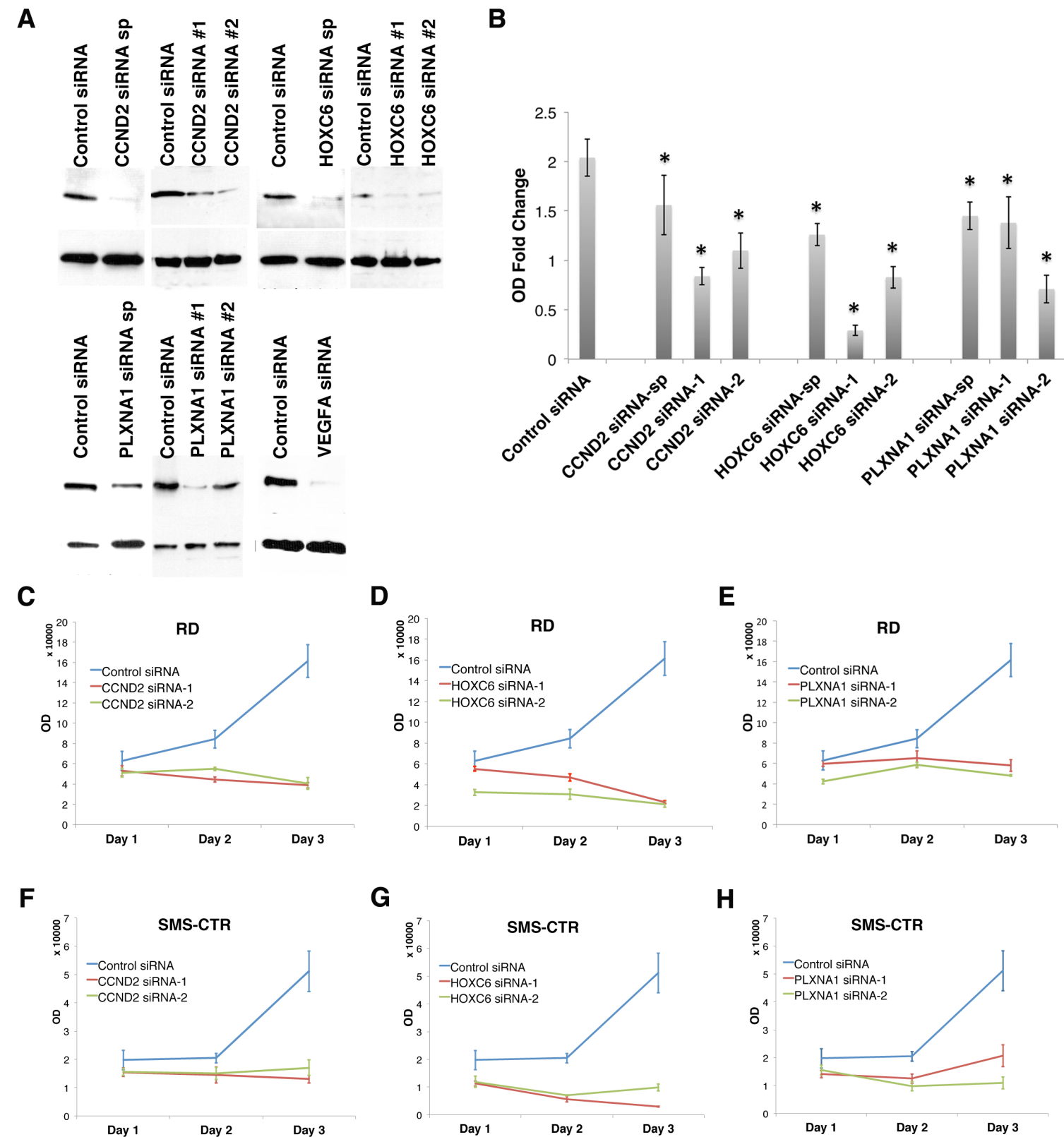

Supplement: Figure S4 — Knockdown of CCND2, HOXC6 and PLXNA1 results in reduced growth of human SMS-CTR and RD cell lines. (A) Western analysis of CCND2, HOXC6 and PLXNA1 knockdowns in SMS-CTR cell line using smart-pool (sp) and two individual gene-specific siRNAs. Bottom row: GAPDH. Percentage knockdown for each siRNA is as follows: CCND2 siRNA-sp, 98%; CCND2 siRNA#1, 57%; CCND2 siRNA#2, 91%; HOXC6 siRNA-sp, 82%; HOXC6 siRNA#1, 88%; HOXC6 siRNA#2, 75%; PLXNA1 siRNA-sp, 77%; PLXNA1 siRNA#1, 94%; PLXNA1 siRNA#2, 54%; VEGFA siRNA, 97%. (B) Summary of cell-titer glo analysis in SMS-CTR cell line. OD fold changes over 3 days for smart pool (sp) and individual siRNAs are shown. Asterisk indicates statistical significance in comparison to control siRNA treatment by Student's t-test (p<0.05). Cell-titer glo assay showing OD change in 3 days for RD (C–E) and SMS-CTR (F–H) cells transfected with two individual gene-specific siRNAs. Each error bar indicates standard deviation of triplicate experiments. (PDF) [file pgen.1003727.s004.pdf]

Fig. S5

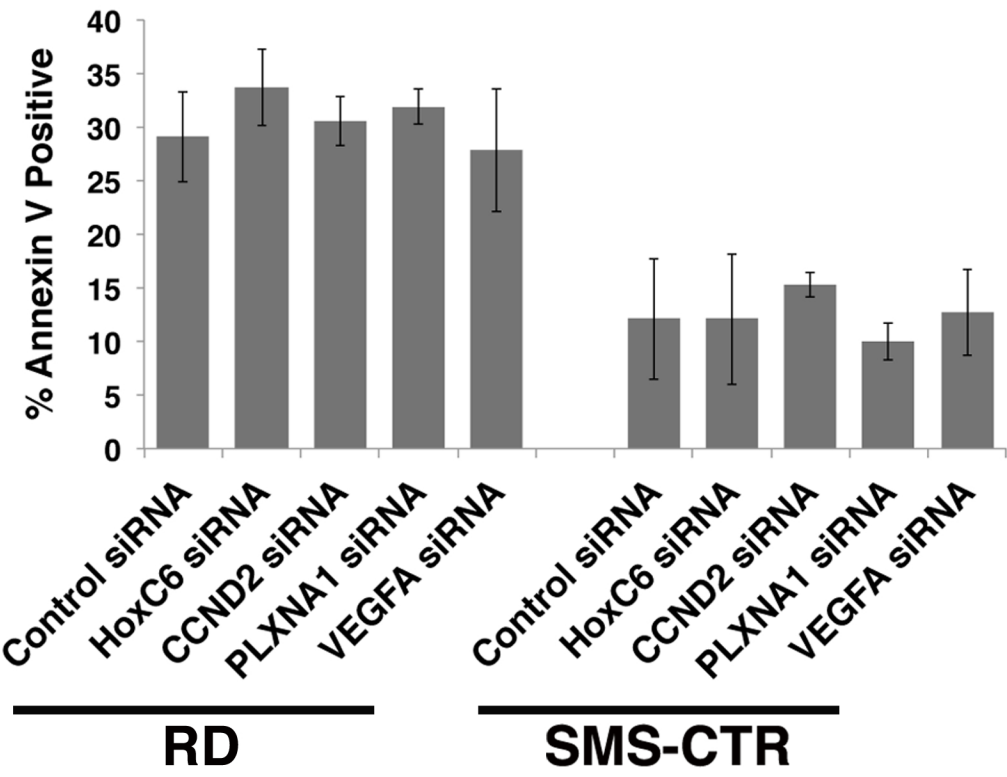

Supplement: Figure S5 — Knockdown of CCND2, HOXC6, PLXNA1 and VEGFA did not affect apoptosis. Annexin V analysis was performed on RD and SMS-CTR cells transfected with siRNA targeted against CCND2, HOXC6, PLXNA1 and VEGFA. Each error bar indicates standard deviation of triplicate experiments. (PDF) [file pgen.1003727.s005.pdf]

**Fig. S6**

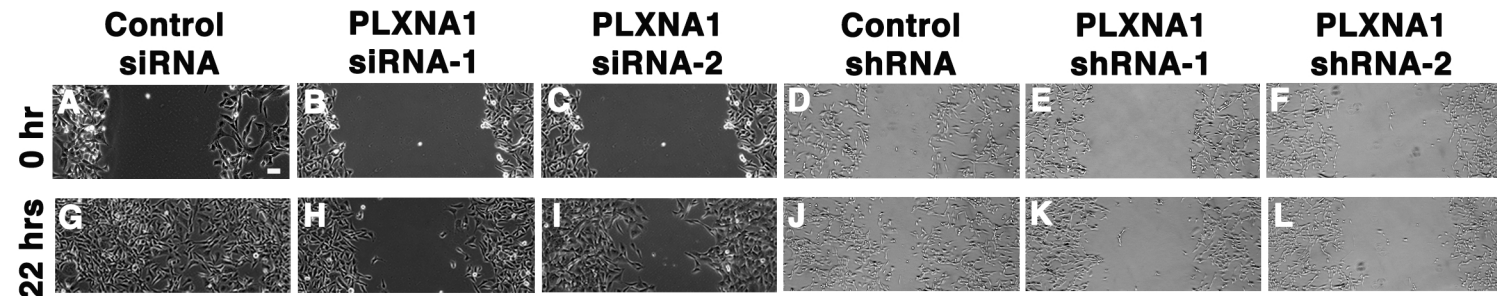

**M**

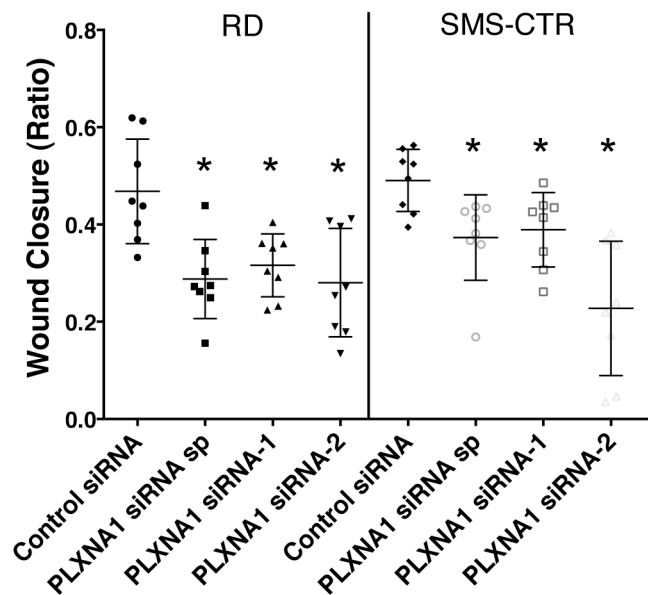

**N**

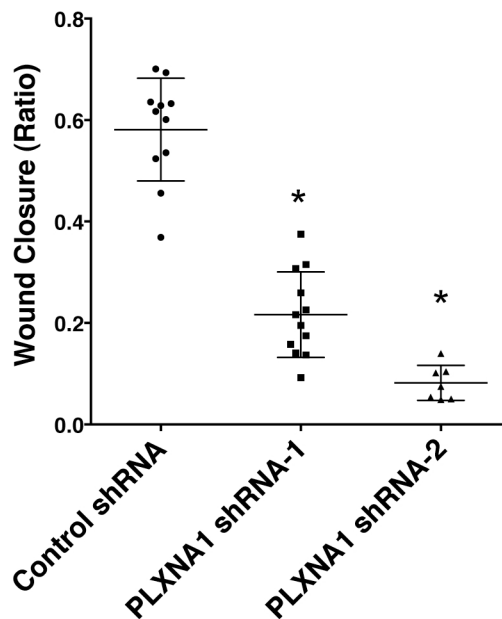

Supplement: Figure S6 — Knockdown of PLXNA1 results in impaired migration in a wound-healing assay. Cells transfected with siRNA or shRNA were allowed to migrate over a scratch wound over 22 hours. Representative images of cells with control siRNA, two gene-specific PLXNA1 siRNAs, control shRNA and two gene-specific shRNAs at 0 hr (A–F) and 22 hrs (G–L) are shown. Scale bar indicates 50 µm. (M) Summary of assessing PLXNA1 knockdown in wound healing assays using two gene-specific siRNAs in RD and SMS-CTR cell lines. Eight random measurements at each time point were made for each siRNA. The ratio of wound closure was determined by the difference in distance migrated over total distance. Each error bar denotes standard deviation. (N) Summary of wound healing assays using two gene-specific PLXNA1 shRNAs. (PDF) [file pgen.1003727.s006.pdf]

**Fig. S7**

***HOXC6***

**Relative Expression**

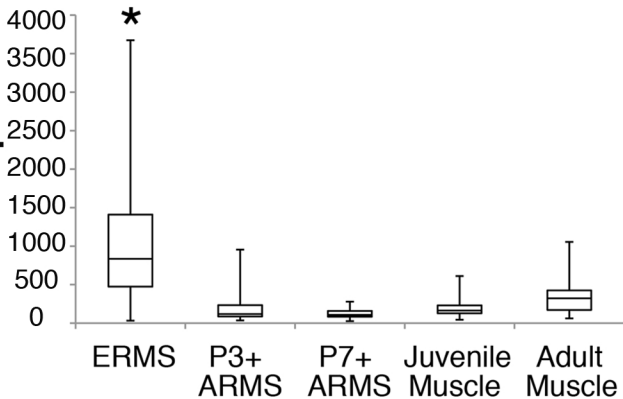

Supplement: Figure S7 — HoxC6 is differentially expressed in ERMS when compared to ARMS and normal muscle. Wisker plot showing relative mRNA expression levels of HOXC6 in human RMS in comparison with juvenile and adult muscle as assessed by microarray gene expression. Asterisk denotes statistical significance based on Student's t-test (p<0.001). PAX3-FKHR+ ARMS (P3+ ARMS), PAX7-FKHR+ ARMS (P7+ ARMS). (PDF) [file pgen.1003727.s007.pdf]

**Fig. S8**

**A**

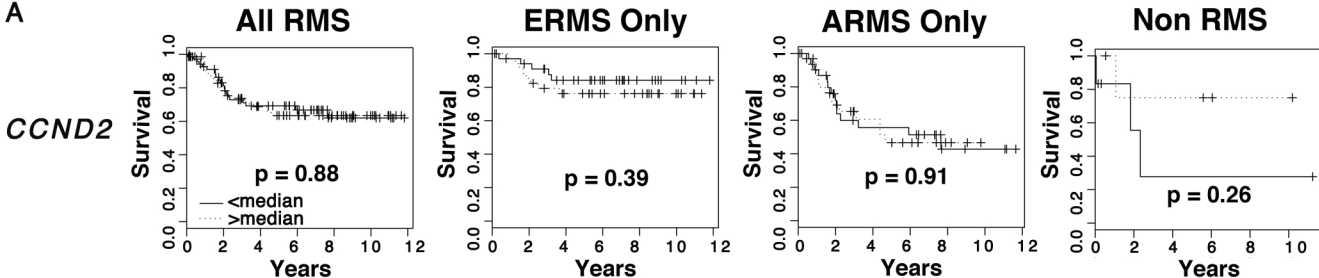

**B**

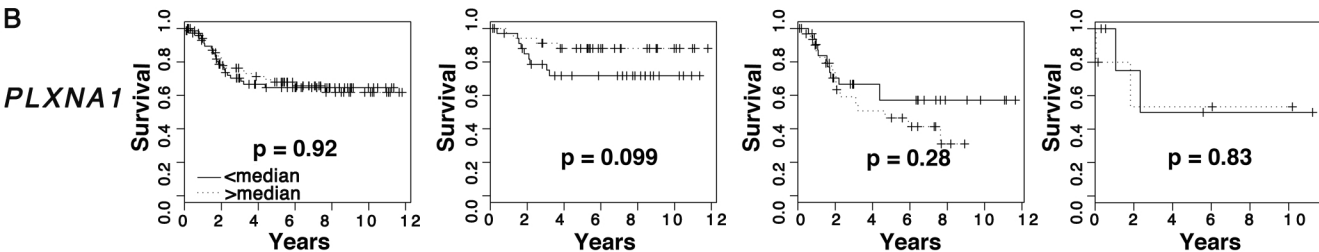

Supplement: Figure S8 — Kaplan-Meier analysis correlating expression levels of CCND2 and PLXNA1 with clinical survival. Kaplan-Meier analysis was completed using microarray data from Davicioni et al (2010) to correlate expression levels of CCND2 and PLXNA1 with clinical survival. Comparison was made in all RMS patients, ERMS patients only, ARMS patients only and non-RMS patients. (A) CCND2. (B) PLXNA1. (PDF) [file pgen.1003727.s008.pdf]

Fig. S9

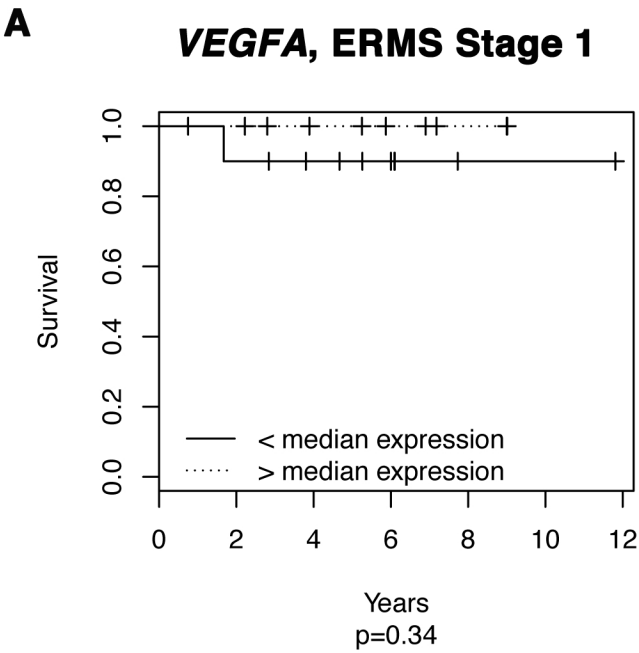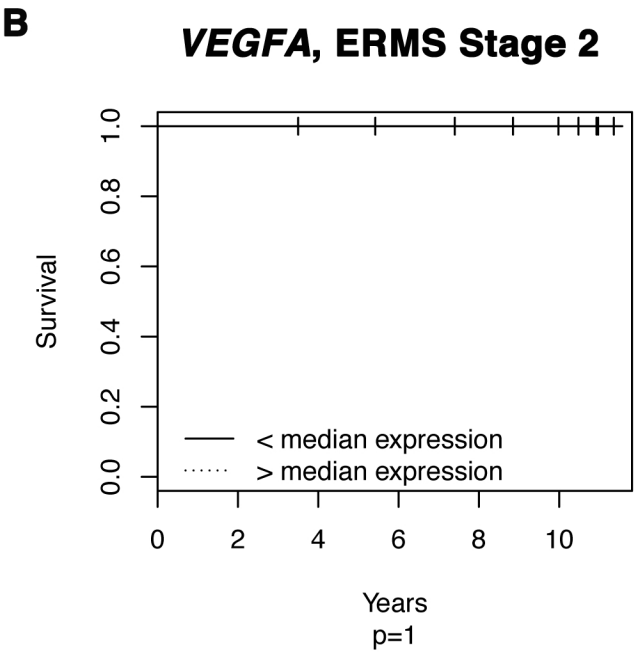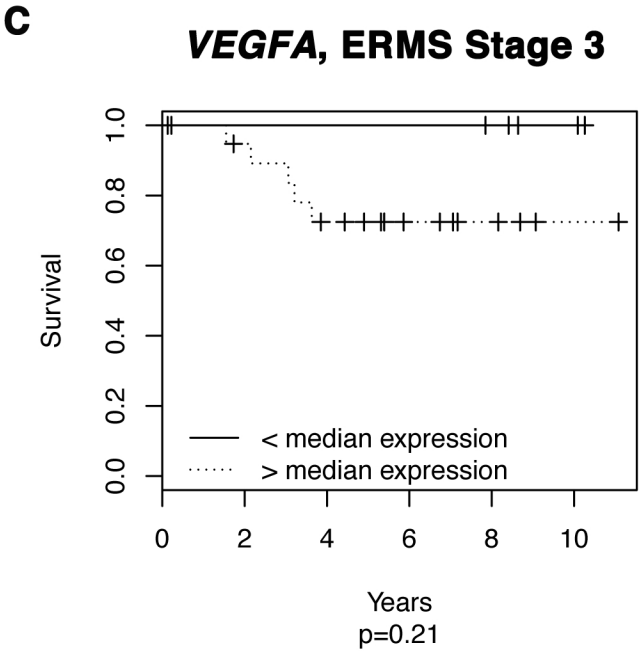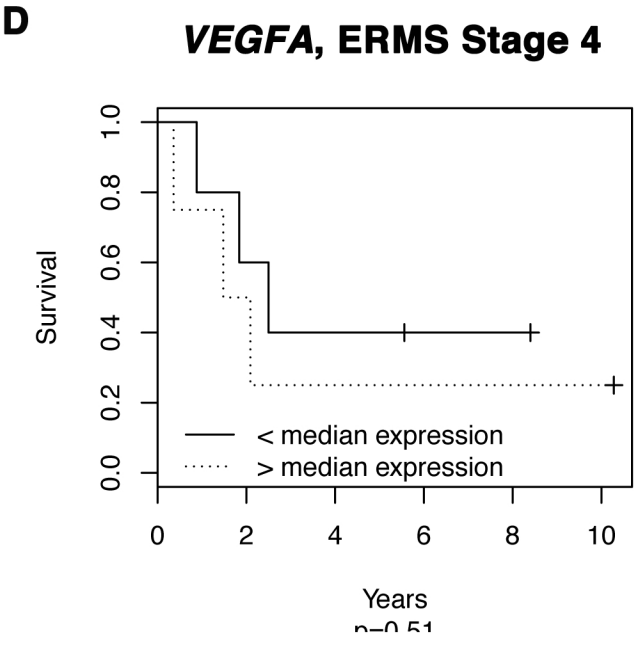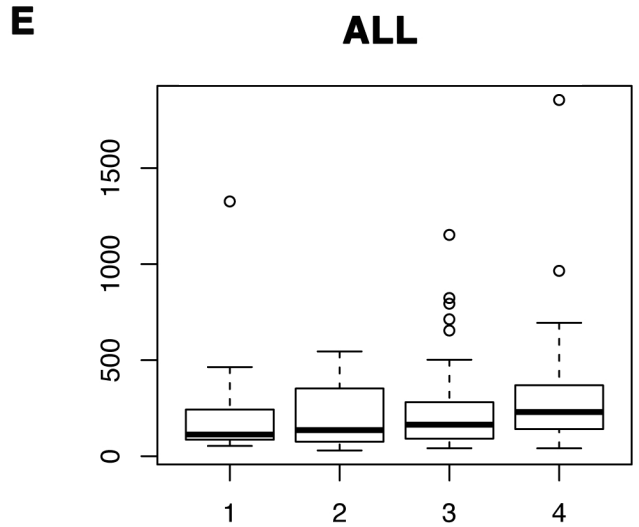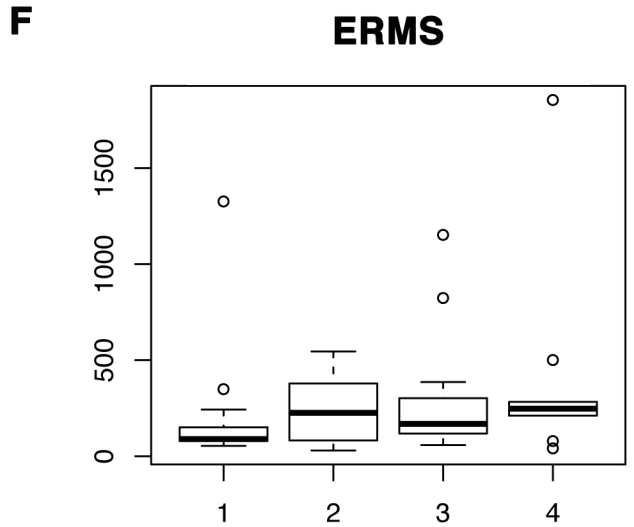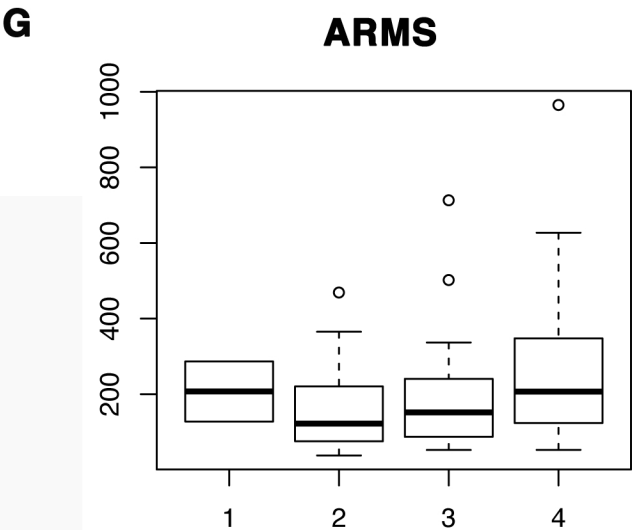

Supplement: Figure S9 — VEGFA transcript expression does not correlate with clinical stage of ERMS. Kaplan-Meier analysis was completed using microarray data from Davicioni et al (2010). (A) Stage 1. (B) Stage 2. (C) Stage 3. (D) Stage 4. Normalized mRNA expression levels of VEGFA across all RMS (E), ERMS (F) and ARMS (G) samples were also assessed in box plots. (PDF) [file pgen.1003727.s009.pdf]
