# Supplementary material for: Cross-Species Array Comparative Genomic Hybridization Identifies Novel Oncogenic Events in Zebrafish and Human Embryonal Rhabdomyosarcoma
Source: PLoS Genet. 2013 Aug 29;9(8):e1003727. doi: 10.1371/journal.pgen.1003727 (PMC3757044; doi:10.1371/journal.pgen.1003727)
Supplement: Table S1 — Summary of zebrafish samples with recurrent regional gains and deletions in array CGH analysis. (PDF) [file pgen.1003727.s010.pdf]

Supplemental Table 1. Summary of Recurrent CNA in Zebrafish ERMS

Chromosome	Start	End	Gain/Loss	Fish with Abberation
1	3221798	3254054	CN Loss	12,13,17,18,19,29,
1	20590042	20649701	CN Gain	14,26,28,30,
1	41652513	41725104	CN Gain	26,28,30,17,
1	54156980	54166600	CN Loss	10,11,12,
2	17238053	17299500	CN Gain	14,18,24,
2	17427930	17437602	CN Gain	14,18,28,
2	27016046	27045160	CN Gain	14,26,28,3,
2	31948129	31969902	CN Gain	14,18,24,26,
2	34139802	34193950	CN Gain	10,11,12,17,18,19,
2	37135899	37232648	CN Gain	14,26,28,
2	44566248	44586182	CN Gain	14,18,26,
2	45978605	45994948	CN Gain	14,18,24,26,
2	48193897	48203995	CN Loss	24,25,28
3	2503226	2521958	CN Loss	11,13,28,
3	20700973	20743658	CN Gain	14,18,24,
3	33203017	33301376	CN Gain	14,21,26,28,30,
3	34639964	34668782	CN Gain	20,21,28,30,
3	40629523	40657635	CN Gain	20,21,30,8,
3	43953554	44000977	CN Loss	12,13,17,18,19,
4	5780206	5817754	CN Gain	14,18,24,
4	6800440	6846728	CN Gain	14,24,26,
4	11623588	11645637	CN Gain	14,18,24,
4	17660064	17677467	CN Gain	10,14,18,
4	23763149	23789590	CN Loss	12,13,17,19,
4	27351280	27374265	CN Gain	14,26,28,
4	27448001	27461211	CN Gain	14,26,28,
4	28728324	28754191	CN Gain	14,26,28,
4	30173128	30195701	CN Gain	26,28,30
4	31953799	31990416	CN Loss	12,13,17,19,
4	34355236	34368819	CN Gain	11,14,18,26,3,30
4	34597856	34623629	CN Gain	26,28,14,
4	34859083	34871362	CN Gain	3,26,28,
4	35402460	35418585	CN Gain	26,28,3,
4	36232929	36278048	CN Gain	14,26,28,
4	37906676	37971177	CN Gain	14,26,28,
4	38389755	38454927	CN Gain	14,26,28,
4	38880655	38906425	CN Gain	14,26,28,
4	39300130	39315363	CN Gain	14,26,28,
4	39465096	39510129	CN Gain	14,26,28,
4	39900059	39938282	CN Gain	14,26,28,
4	40186811	40209617	CN Gain	14,26,28,
4	40465659	40486890	CN Gain	14,26,28,
4	40638255	40673728	CN Gain	14,26,28,
4	40909122	40925248	CN Gain	28,26,14,
4	41051080	41080872	CN Gain	14,18,24,26,
4	41127164	41145032	CN Gain	24,26,28,30,
4	41228823	41257936	CN Gain	14,26,28,
4	41534801	41548234	CN Gain	14,26,18,
4	42254004	42286196	CN Gain	28,26,14,
4	42515234	42623266	CN Gain	14,26,28,
4	43263429	43323061	CN Gain	14,26,28,
4	44043735	44056752	CN Gain	14,26,28,
4	44488109	44508357	CN Gain	14,26,28,
4	44824299	44834004	CN Gain	14,26,28,

4	45120026	45169377	CN Gain	14,26,28,
4	45262932	45306950	CN Gain	14,26,28,30,3,
4	45533435	45544156	CN Gain	26,28,30
4	46178863	46201288	CN Loss	12,13,17,19,
4	46972250	46985415	CN Loss	21,20,29
4	46985415	47005043	CN Gain	14,26,28,
4	47394628	47445453	CN Gain	14,26,28,30,
4	48534666	48560466	CN Gain	18,26,3,
4	48658853	48723329	CN Gain	14,26,28,
4	49036178	49065173	CN Gain	14,26,28,
4	49355428	49374779	CN Gain	14,26,28,
4	50642702	50667476	CN Gain	14,26,28,
4	51300100	51322676	CN Gain	14,26,28,
4	51338919	51384039	CN Gain	14,28,26,
4	51419426	51438265	CN Gain	14,28,26,
4	51880598	51890951	CN Gain	14,26,28,
4	51917144	51932472	CN Gain	14,26,28,
4	52564300	52577200	CN Gain	26,28,14
4	52702980	52715906	CN Gain	11,28,30,
4	52818357	52861029	CN Gain	11,14,26,
4	52921097	52948716	CN Gain	14,30,3,
4	53200162	53214318	CN Loss	13,24,25,
4	53517319	53546310	CN Gain	14,26,28,
4	53687368	53715623	CN Gain	14,26,28,
4	53980073	53989755	CN Gain	14,26,18,
4	54207440	54225174	CN Gain	14,28,26,
4	54866918	54892749	CN Gain	26,28,14,
4	55960037	55970128	CN Gain	8,3,26,28,
4	56166923	56202543	CN Gain	14,26,28,
4	56302665	56384609	CN Gain	14,26,28,
4	56392374	56418636	CN Gain	14,26,28,11,3,
4	56772167	56841279	CN Gain	14,26,28,
4	57024503	57056161	CN Gain	14,26,18
4	57430456	57459628	CN Loss	12,13,24,25,
4	58340141	58388263	CN Gain	26,28,14,
4	59027204	59093312	CN Gain	14,26,30,
4	59375508	59478083	CN Gain	14,26,28,
4	59524991	59543199	CN Gain	14,26,28,
4	59707841	59739956	CN Gain	14,26,28,
4	59833829	59853089	CN Gain	14,26,28,
4	60207553	60236577	CN Gain	14,26,28,
4	61942602	61990974	CN Gain	14,26,28,
4	62365074	62403774	CN Gain	14,26,28,30,
4	62632720	62684354	CN Gain	14,26,28,
4	63171355	63187450	CN Gain	14,26,28,
4	63706236	63722799	CN Loss	28,29,3,
4	63929200	63957892	CN Gain	26,28,14,
4	63963926	63983784	CN Gain	26,28,14
4	65139381	65196596	CN Gain	14,26,28,
4	67305807	67331577	CN Gain	14,26,28,
4	69485876	69498805	CN Gain	14,18,28,26
4	69756455	69805064	CN Gain	14,26,28,
4	69847029	69864036	CN Gain	14,26,28,
5	16324979	16335308	CN Gain	14,18,19,21,
5	21568256	21581729	CN Gain	26,28,30,3,
5	41399379	41421923	CN Gain	10,14,26,
5	42153260	42289784	CN Gain	10,11,14,16,18,24,25,30,

5	47185001	47209187	CN Gain	10,11,14,18,28,
5	47320448	47344639	CN Gain	11,14,18,26,28,
5	53973663	53984910	CN Gain	28,26,14,
5	54205465	54235180	CN Gain	14,28,26,
5	61526574	61544525	CN Loss	13,17,19,
5	72528667	72606093	CN Loss	12,13,17,19,
6	1574030	1584371	CN Gain	24,25,18,
6	18070555	18105205	CN Gain	14,26,28,3,
6	38922551	39022526	CN Gain	11,14,18,26,28,30,3,
6	48813243	48871674	CN Gain	14,20,26,30,
6	52801907	52845160	CN Gain	11,14,26,28,30,3,
7	3402276	3422438	CN Loss	17,13,12,
7	3483329	3488624	CN Loss	17,13,12,
7	5857625	5866298	CN Gain	14,18,24,
7	16859826	16877024	CN Loss	24,25,28
7	39993687	40009121	CN Gain	14,18,24,
7	41960604	41989042	CN Gain	14,24,26,
7	52883579	52909380	CN Gain	18,26,28
7	57387287	57537253	CN Loss	12,17,19
7	61607200	61629775	CN Gain	18,26,28,
7	62623076	62665028	CN Gain	14,26,28,
7	74620223	74639387	CN Gain	11,14,18,26,
8	1422315	1487518	CN Gain	11,14,26,28,30,3,
8	8545546	8562372	CN Gain	14,18,26,28,3,
8	20318310	20330726	CN Gain	14,24,26,
8	22798296	22820155	CN Loss	10,11,13,
8	27783182	27850729	CN Gain	14,18,26,28,
8	32408049	32440568	CN Gain	14,18,26,30,
8	37300373	37313306	CN Gain	14,28,26,
8	37433611	37442420	CN Loss	11,12,24,
8	37535800	37564990	CN Gain	14,26,28,
8	37661578	37698661	CN Loss	12,13,17,
8	38013130	38035673	CN Gain	26,28,30,
8	38135654	38190475	CN Gain	14,20,26,28,30,8,3,
8	50517920	50525046	CN Loss	28,17,13,
8	50532248	50545675	CN Loss	10,13,28,
9	16632326	16685751	CN Gain	20,21,26,29,30,8,
9	23597420	23614991	CN Gain	14,18,24,
9	23706285	23727962	CN Gain	26,28,30,
9	42992483	43117960	CN Gain	10,11,12,14,16,17,18,24,25,26,28,30,3,
9	43149720	43262656	CN Gain	18,28,3,14,24
9	43272584	43312677	CN Gain	10,11,18,24,25,26,28,30,3,
9	44940822	44963697	CN Gain	26,28,3,
10	18469428	18523979	CN Gain	14,18,24,
10	35806743	35862551	CN Gain	11,14,16,28,30,
12	3257277	3276616	CN Gain	14,26,28,
12	12938849	12957084	CN Gain	20,21,26,30,
12	28044434	28065707	CN Loss	12,13,17,
12	40244886	40264212	CN Gain	14,26,28,
13	2780207	2792647	CN Loss	12,24,25,
13	33784718	33801633	CN Gain	11,26,28,
13	45210517	45289299	CN Gain	11,14,16,26,28,30,
14	2525201	2580026	CN Gain	24,26,28
14	2739663	2783205	CN Gain	14,18,28,
14	21281798	21388229	CN Loss	12,13,17,18,19,29,
14	28522122	28538163	CN Gain	14,26,28,
15	186438	238755	CN Gain	11,14,18,26,28,30,3,

15	3366927	3379370	CN Gain	14,18,26,28,
15	7153543	7165848	CN Gain	14,24,25,26,
15	9916898	9966892	CN Gain	11,26,28,3,
15	10790873	10807804	CN Gain	18,26,28,
15	18476190	18489416	CN Gain	18,26,28,
15	20951335	20971792	CN Gain	26,3,28
16	8616099	8709682	CN Gain	10,11,14,18,26,28,30,3,
16	18979568	18995151	CN Gain	14,18,24,
16	21336252	21420474	CN Gain	14,20,21,26,28,29,30,8,
16	25757734	25770374	CN Gain	14,26,28,
16	25848403	25883875	CN Loss	12,13,17,19,
16	27015356	27063074	CN Gain	14,26,28,30,
17	20985791	21044503	CN Gain	14,18,26,28,
17	25451411	25467401	CN Gain	14,18,24,
18	8998789	9056561	CN Gain	11,14,16,26,28,30,
18	9743339	9759088	CN Gain	14,18,24,26,
18	45084174	45098051	CN Gain	14,24,26,28,
19	3289509	3299415	CN Loss	11,24,25,
19	7609792	7633205	CN Loss	13,17,29,
19	12558378	12571589	CN Gain	14,18,26,
19	14042083	14061438	CN Gain	14,18,24,
19	18569575	18598599	CN Gain	20,26,28,30,
19	34427807	34440105	CN Gain	14,18,24,
19	36758799	36816014	CN Gain	14,18,26,28,
19	41834730	41902454	CN Gain	11,14,26,28,30,3,
20	9036476	9104203	CN Gain	14,18,26,
20	11487477	11508405	CN Gain	20,21,28,29,30,
20	23975025	24002706	CN Gain	14,18,24,26,
20	25403030	25468401	CN Gain	11,14,18,26,28,30,
20	30360472	30403453	CN Gain	10,11,18,19,24,
20	37417560	37438424	CN Gain	14,18,24,
20	46027203	46049804	CN Gain	14,26,28,
20	50222948	50248779	CN Gain	14,24,25,26,
21	10851248	10884400	CN Gain	14,26,28,30,3,
21	11061775	11094027	CN Gain	14,26,28,
21	13964277	13977719	CN Gain	26,28,30,3,
21	16520939	16534601	CN Gain	11,14,18,26,28,
21	19294011	19363369	CN Gain	14,18,24,26,30,3,
21	19524849	19592410	CN Gain	14,18,26,30,3,
21	28039786	28176855	CN Loss	12,13,17,18,19,
21	31946878	31995278	CN Gain	24,26,28,
21	46585152	46610954	CN Gain	11,14,16,18,24,26,30,
22	30166680	30192003	CN Gain	14,26,28,
23	2460170	2546633	CN Gain	11,14,16,17,18,24,25,26,28,30,
23	35111111	35140132	CN Gain	14,18,24,16,
23	42560287	42573707	CN Gain	28,26,30,3,
24	37907257	37963745	CN Loss	12,24,25,
25	8143188	8152950	CN Gain	10,11,12,13,14,16,17,18,19,20,21,24,25,26,28,29,30,8,3
25	9421012	9436809	CN Loss	25,24,13,
25	9471213	9487142	CN Loss	12,13,25,
25	9543462	9557124	CN Loss	13,24,25,
25	11881471	11894564	CN Gain	14,18,26,
25	14304416	14320461	CN Gain	14,18,24,
25	26255303	26270747	CN Gain	14,18,24,
25	26345084	26361179	CN Gain	14,18,24,
25	27253828	27329135	CN Gain	14,18,26,
25	36472057	36478494	CN Loss	11,13,12,
