# Supplementary material for: Cross-Species Array Comparative Genomic Hybridization Identifies Novel Oncogenic Events in Zebrafish and Human Embryonal Rhabdomyosarcoma
Source: PLoS Genet. 2013 Aug 29;9(8):e1003727. doi: 10.1371/journal.pgen.1003727 (PMC3757044; doi:10.1371/journal.pgen.1003727)
Supplement: Table S2 — Summary of clinical information and immunohistochemical staining results of human RMS samples. (PDF) [file pgen.1003727.s011.pdf]

Supplemental Table 2: Clinical information and summary of immunohistochemical staining results of human RMS samples.

Clinical Data					IHC Staining			
**Sample ID**	**Age**	**Sex**	**Site**	**Mestatasis?**	**CCND2**	**HOXC6**	**PLXNA1**	**VEGFA**
**ARMS 1**	23	M	left nasal cavity, masillary sinus, orbit and frontal sinus	No	2W	0	3S	1W
**ARMS 2**	21 months	F	right thigh	No	2W	3S	3S	1W
**ARMS 3**	42	F	right posterior neck	Yes	2W	1W	3S	0
**TMA ARMS 1**	10	M	Striated muscle		3S	0	1W	3W
**TMA ARMS 2**	10	M	Striated muscle		3S	0	0	1W
**TMA ARMS 3**	48	M	Soft tissue		0	0	3W	2W
**TMA ARMS 4**	48	M	Soft tissue		0	0	3W	2W
**ERMS 1**	5	M	head and neck		3S	1S	0	3S
**ERMS 2**	12	M	paratesticular		1S	1W	3S	0
**ERMS 3**	37	F	right shoulder	Yes	3S	3S	0	3S
**ERMS 4**	23	F	skull base	Yes	1S	N/A	3S	1W
**ERMS 5**	25	M	parapharyngeal space	No	3S	3S	3S	1W
**TMA ERMS 1**	21	M	Retroperitoneum	No	3S	3S	3S	3W
**TMA ERMS 2**	21	M	Retroperitoneum	No	3S	3S	3S	3W
**TMA ERMS 3**	16	M	Testis	No	3S	3S	1W	0
**TMA ERMS 4**	16	M	Testis	No	3S	3S	1W	1W
**TMA ERMS 5**	18	M	Tongue	No	1W	1W	2W	0
**TMA ERMS 6**	18	M	Tongue	No	1W	1W	2W	1W
**TMA ERMS 7**	40	F	Abdominal cavity	No	1W	1W	3W	0
**TMA ERMS 8**	40	F	Abdominal cavity	No	1W	0	3W	0
**TMA ERMS 9**	23	F	Pelvic cavity	No	1W	0	3W	0
**TMA ERMS 10**	23	F	Pelvic cavity	No	1W	0	3W	0
**TMA ERMS 11**	40	M	Soft tissue	No	0	0	3W	0
**TMA ERMS 12**	40	M	Soft tissue	No	0	0	3W	0
**TMA ERMS 13**	49	M	Testis	No	0	3W	3W	2W
**TMA ERMS 14**	49	M	Testis	No	0	3W	3W	3W
**Fetal Muscle**					0	0	0	0
**Adult Muscle**					0	0	0	0

S: Strong
W: Strong
0: negative staining.
1-3: positive staining
1: <25% of tumor cells positive
2: 25-75% of tumor cells positive
3: >75% of tumor cells positive
