# Supplementary material for: Cross-Species Array Comparative Genomic Hybridization Identifies Novel Oncogenic Events in Zebrafish and Human Embryonal Rhabdomyosarcoma
Source: PLoS Genet. 2013 Aug 29;9(8):e1003727. doi: 10.1371/journal.pgen.1003727 (PMC3757044; doi:10.1371/journal.pgen.1003727)
Supplement: Table S3 — Summary of clinical information and immunohistochemical staining results of RMS samples in the Children's Oncology Group tissue microarray. (PDF) [file pgen.1003727.s012.pdf]

Supplemental Table 3
COG TMA Clinical Information and VEGFA Immunohistochemical Staining

Sample ID	Gender	Years	Months	Site	VEGFA Staining
1	M	5	8	BUCCAL MUCOSA, INTERNAL CHEEK	3
2	M	16	5	SHOULDAR MASS	2
3	M	1	11	SCROTUM, RIGHT	3
4	F	6	2	RETROPARATONEUM	0
5	M	6	1	RIGHT TESTIS	2
6	F	2	4	GLUTEUS MAXIMUS, RIGHT	3
7	M	6	10	TESTICLE, RIGHT	3
8	M	15	8	TESTIS, LEFT	2
9	M	1	5	ABDOMEN	3
10	M	17	0	TESTICLE, LEFT	2
11	F	6	1	RETROPERITONEAL MASS	3
12	M	5	9	ARM, RIGHT/FOOT, RIGHT	2
13	M	6	2	BLADDER	0
14	M	6	2	JAW, CAVITY LEFT	3
15	M	11	4	PARATESTICULAR	1
16	M	4	8		3
17	M	5	5	RETROPERITONEAL	3
18	M	15	8	PARATESTICULAR	3
19	M	6	4	TESTIS NOS	3
20	M	1	11	BLADDER/PROSTATE	0
21	M	14	2	TESTIS	2
22	F	4	9	PERITONEUM	3
23	F	5	6	PELVIS	0
24	M	5	3	ORBIT,RIGHT	3
25	M	5	2	ABDOMEN	2
26	M	3	5	ABDOMEN	0
27	F	2	8	BLADDER	3
28	F	7	4	PAROTID GLAND	2
29	F	3	8	UNKNOWN	2
30	F	1	9	NASOPHARYNX	2
31	M	5	7		3
32	M	7	3	TESTICULAR	3
33	M	2	5	NASOPHARYNGEAL	3
34	M	14	5	TESTICLE	0
35	F	11	11	RETROPERITONEUM	0
36	M	11	1	TESTICLE	3
37	F	3	10	BUTTOCK, RIGHT	3
38	M	8	8	TESTICULAR, RIGHT	3

0: negative staining.
1-3: positive staining
1: <25% positive
2: 25-75% positive
3: >75% positive
