# Supplementary material for: Cross-Species Array Comparative Genomic Hybridization Identifies Novel Oncogenic Events in Zebrafish and Human Embryonal Rhabdomyosarcoma
Source: PLoS Genet. 2013 Aug 29;9(8):e1003727. doi: 10.1371/journal.pgen.1003727 (PMC3757044; doi:10.1371/journal.pgen.1003727)
Supplement: Table S4 — Summary of shRNA sequences and quantitative primers used in array CGH validation studies. (PDF) [file pgen.1003727.s013.pdf]

Supplemental table 4

Sequences of shRNA used in human cell line knockdown studies and primer sequences used for copy number validation in zebrafish aCGH.

PLXNA1 shRNA-1: GCCCACAATTAGCTGTCCTTT

PLXNA1 shRNA-2: CGCAGTGAACCGCATCTATAA

Scrambled shRNA: TCCTAAGGTTAAGTCGCCCTCG

Primers	Sequence
z-plxna-1-F	CGAAGGACACGTACTGAACCTGCA
z-plxna-1-R	TCAGGACATATCTCAGCCCAGGCA
z-plxna-2-F	ACGAAGGACACGTACTGAACCTGC
z-plxna-2-R	CAGGACATATCTCAGCCCAGGCA
z-ptch2-1-F	CCTTGGGGATGTGCCCAGCG
z-ptch2-1-R	GTCTGCCCTTGCCTGCCCAG
z-ptch2-2-F	ACAAAGCCAGGCCCCCTCCA
z-ptch2-2-R	GCAGGGAGGCTCCGCCTACT
z-tmeff2a-1-F	ACGCACCTACGGTGCTTGGC
z-tmeff2a-1-R	TTGCGGAGTTTCGGCGCACT
z-limk1-1-F	TATAGCGCCACCATGCGGCC
z-limk1-1-R	CGACGCACCGTTTTCCCGCTA
z-limk1-2-F	CGCCCCCTAGTGGCCGATTT
z-limk1-2-R	ACGCACCGTTTTCGCGCTACA
z-ccnd-2a-1-F	CCGTTTCCTCCCACACCGGC
z-ccnd-2a-1-R	GCTAGCGGCAGAGCAGGACG
z-ccnd-2a-2-F	AGGCCTGCACAGGAACACGC
z-ccnd-2a-2-R	GGCCGGTGTGGGAGGAAACG
z-HoxB4-1-F	CCACGCTCGGGGGAACGTAC
z-HoxB4-1-R	TGCCGGGTGTCCTGTCGCTA
z-HoxB4-2-F	CACGCTCGGGGGAACGTACA
z-HoxB4-2-R	GCACGGAGGGTTGCGTGCAT
z-HoxC8D8-1-F	CGGCGCTTGCGTGTGAGGTA
z-HoxC8D8-1-R	CGCGCGCACACCAAGTTGTG
z-HoxC8D8-2-F	GGTTGTACCCGTGAGTGCTT
z-HoxC8D8-2-R	CGCCTCCTCTTGTAGAATGC
z-HoxA3-1-F	GGCATTAACAGTCCAATGGGATGCT
z-HoxA3-1-R	CGCGTGATGACGGTCGCTGA
z-HoxA3-2-F	ACATTAAGCTGGGTGGATGC
z-HoxA3-2-R	CCGAAAAACGTATGGTGCTT
z-chr2-loss-aCGH-1-F	CGCCTCTGACCCCAGCAAGC

z-chr2-loss-aCGH-1-R	GTGGAATGCCAGGGGGCACC
z-chr2-loss-aCGH-2-F	GGTGCCCCCTGGCATTCCAC
z-chr2-loss-aCGH-2-R	GCTTGCTGGGGTCAGAGGCG
z-chr16-loss-aCGH-1-F	GTGCCCATTGGCCCTCCACC
z-chr16-loss-aCGH-1-R	GGGCAGCTTGCTGGGGTCAG
z-chr16-loss-aCGH-2-F	GTGCCCATTGGCCCTCCACC
z-chr16-loss-aCGH-2-R	GGCCCCTGGACAGCTTGCTG
z-chr5-loss-aCGH-1-F	CGCTGGCCCTTCACCAAGGG
z-chr5-loss-aCGH-1-R	GAGGGCAGGGCGTCACCCTA
z-chr5-loss-aCGH-2-F	AGGCCCTAGGGTGCGGTCTG
z-chr5-loss-aCGH-2-R	CTAGGTGGAGGGCAGGGCGT
