# Supplementary material for: Cross-Species Array Comparative Genomic Hybridization Identifies Novel Oncogenic Events in Zebrafish and Human Embryonal Rhabdomyosarcoma
Source: PLoS Genet. 2013 Aug 29;9(8):e1003727. doi: 10.1371/journal.pgen.1003727 (PMC3757044; doi:10.1371/journal.pgen.1003727)
Supplement: Figure S3 — Knockdown of candidate genes and inhibition of VEGFA activity in ERMS cell lines. (A) ELISA to assess VEGFA protein level in growth media of transfected cells. (B) qRT-PCR to assess knockdown of mRNA levels of candidate genes. (Error bars indicate standard deviation of each triplicate within each experiment). Asterisks denote significant differences with p<0.003 and p<0.008, respectively within each comparison. (PDF) [file pgen.1003727.s003.pdf]

Fig. S3

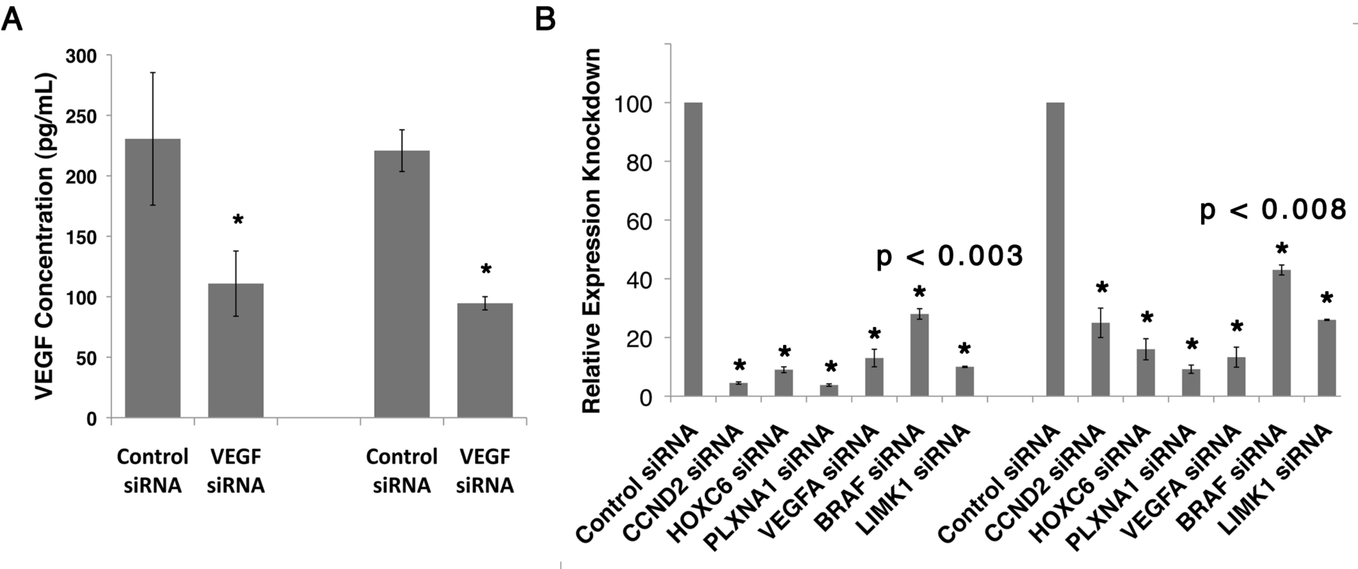
A
VEGF Concentration (pg/mL)
300
250
200
150
100
50
0
*
*
Control siRNA
VEGF siRNA
Control siRNA
VEGF siRNA
B
Relative Expression Knockdown
100
80
60
40
20
0
p < 0.003
p < 0.008
Control siRNA
CCND2 siRNA
HOXC6 siRNA
PLXNA1 siRNA
VEGFA siRNA
BRAF siRNA
LIMK1 siRNA
Control siRNA
CCND2 siRNA
HOXC6 siRNA
PLXNA1 siRNA
VEGFA siRNA
BRAF siRNA
LIMK1 siRNA
